# Succession of biofilm communities responsible for biofouling of membrane bio-reactors (MBRs)

**DOI:** 10.1371/journal.pone.0179855

**Published:** 2017-07-07

**Authors:** Jinxue Luo, Pengyi Lv, Jinsong Zhang, Anthony G. Fane, Diane McDougald, Scott A. Rice

**Affiliations:** 1 School of Biological Sciences, Nanyang Technological University, Singapore, Singapore; 2 Advanced Environmental Biotechnology Centre, Nanyang Environment and Water Research Institute, Nanyang Technological University, Singapore, Singapore; 3 Research Center for Eco-Environmental Sciences, Chinese Academy of Sciences, Beijing, China; 4 School of Chemical and Environmental Engineering, China University of Mining and Technology, Beijing, China; 5 Singapore Membrane Technology Centre, Nanyang Environment and Water Research Institute, Nanyang Technological University, Singapore, Singapore; 6 Singapore Centre for Environmental Life Sciences Engineering, Nanyang Technological University, Singapore, Singapore; 7 The ithree Institute, The University of Technology Sydney, Sydney New South Wales, Sydney, Australia; 8 Centre for Marine Bio-Innovation, School of Biological, Earth and Environmental Sciences, The University of New South Wales, Sydney, Australia; University of Notre Dame, UNITED STATES

## Abstract

Biofilm formation is one of the main factors associated with membrane biofouling in membrane bioreactors (MBRs). As such, it is important to identify the responsible organisms to develop targeted strategies to control biofouling. This study investigated the composition and changes in the microbial communities fouling MBR membranes over time and correlated those changes with an increase in transmembrane pressure (TMP). Based on qPCR data, bacteria were the dominant taxa of the biofilm (92.9–98.4%) relative to fungi (1.5–6.9%) and archaea (0.03–0.07%). NMDS analysis indicated that during the initial stages of operation, the biofilm communities were indistinguishable from those found in the sludge. However, the biofilm community significantly diverged from the sludge over time and ultimately showed a unique biofilm profile. This suggested that there was strong selection for a group of organisms that were biofilm specialists. This pattern of succession and selection was correlated with the rapid increase in TMP, where bacteria including Rhodospirillales, Sphingomonadales and Rhizobiales dominated the biofilm at this time. While most of the identified fungal OTUs matched *Candida* sp., the majority of fungal communities were unclassified by 18S rRNA gene sequencing. Collectively, the data suggests that bacteria, primarily, along with fungi may play an important role in the rapid TMP increase and loss of system performance.

## Introduction

In recent years, membrane bioreactors (MBRs), a water recycling technology integrating activated sludge mediated nutrient removal and membrane filtration in a single system, have attracted increased attention and have been widely applied in wastewater treatment plants [1,2]. This technology has many advantages over the traditional wastewater treatment plant design, such as reducing the hydraulic retention time and the reactor volume, reduced activated sludge biomass production and a higher quality of effluent [3]. Despite its many advantages, biofouling is a critical drawback in MBRs, limiting the wider implementation of the technology [4]. Biofouling is caused by the deposition of microorganisms and microbial products, e.g. polysaccharides and proteins, on the membrane surface [5,6]. In this process, the microbes attach and grow, forming a biofilm on the membrane, clogging the membrane pores and thus increasing the required applied pressure across the membrane, the transmembrane pressure (TMP), and finally affecting the MBR performance [7].

Microorganisms are considered to be one of the most important factors in the biofouling process [6,8]. The Beta-proteobacteria and Bacteroidetes were reported to comprise the majority of the biofilm community in microfiltration [9,10] and nanofiltration MBRs [7]. In contrast, the Gamma-proteobacteria and Actinobacteria were found to be the dominant bacteria in biofilms in both the initial and late fouling stages in other submerged MBR systems [11,12]. Interestingly, the Bacteroidetes and Firmicutes displayed higher abundances in the biofilms than in the activated sludge (Lim *et al*. 2012), while others (Zhang *et al*. 2014) found the Gammaproteobacteria (represented by *Pseudomonas* and *Aeromonas*) preferentially form biofilms on the membranes. Thus, there is no consensus on the dominant bacteria in biofilms and whether there is a correlation between the increased TMP and specific microorganisms.

In addition to bacteria, fungi are also widely reported to be present in MBRs, where they have been found in the anoxic and aerobic sludge as well as in special purpose MBR systems, such as those designed to remove phenol or azo dyes [13,14]. While to date, few studies have focused on the role of fungi in the biofouling process [15], it is clear that a wide spectrum of fungi have the ability to form biofilms, including *Candida* sp. [16], *Saccharomyces* sp. [17], *Cryptococcus* sp. [18] and *Aspergillus* sp. [19]. Additionally, the fungi can also form biofilms in combination with bacteria. For example, investigation of the microbial biofilm community on sandstone in a Bayon temple in Cambodia showed that the microbial community was composed of bacteria, fungi, Metazoa and Alveolata [20]. This study also revealed that the fungal community consisted of Basidiomycota, Ascomycota and Chytridiomycota in both fresh and established biofilms. Not surprisingly, fungi have been shown to be a significant component of activated sludge, where *Geotrichum*, *Penicillium* and yeast can be prevalent in the both anoxic and aerobic sludge [21]. Additionally, keratinophilic fungi, including *Chrysosporium* spp., *Microsporum* spp., *Trichophyton* spp. and *Aspergillus* spp., were also found in the anoxic and aerobic sludge [22]. Thus, it is highly possible that fungi contribute to MBR fouling and biofouling control strategies need to consider the microbial community as a whole rather than focus only on bacteria until a better understanding of the key drivers of fouling are better defined.

In this study, the microbial communities in the biofilm on membranes and in the activated sludge in the aerobic tanks of MBR were characterized through metagenomic sequencing of the 16S and 18S rRNA genes. The relative proportions of the two kingdoms were also determined by qPCR to determine if the biofilm was dominated by fungi or bacteria. The correlation of the bacterial and fungal communities and the TMP increase were determined here with the aim of trying to determine if specific community members were correlated with the TMP increase.

## Materials and methods

### MBR reactors and operation

Three independent experiments using two different types of laboratory-scale MBRs, internal and external submerged MBR [23], were conducted to compare biofilm development with MBR performance. Experiments 1 and 2, which were performed in parallel, utilized internal submerged MBRs, while experiment-3 used an external submerged MBR (S1 Fig). The membrane modules for all three MBRs consisted of 100 pieces of hollow fiber (HF) PVDF membranes (ZeeWeed, GE) and were assembled as a “curtain” style module. The average length of each hollow fiber membrane was 10 cm and the total area for each membrane module was 565 cm^2^. One end of the membrane was sealed and hung down into the sludge tank (free ends). The other ends of the membranes were open and sealed into a collection chamber that was linked to a suction pump (S1 Fig).

All three experiments were operated using synthetic wastewater with a total organic carbon (TOC) concentration of 200 mg/L. The synthetic wastewater was composed of glucose (320 mg/L), beef extract (60 mg/L), peptone (80 mg/L), KH_2_PO_4_ (7 mg/L), MgSO_4_•7H_2_O (14 mg/L), FeSO_4_•7H_2_O (7.3 mg/L) and sodium acetate (90 mg/L). For each experiment, fresh activated sludge was collected from the Ulu Pandan wastewater treatment plant in Singapore and acclimated in artificial synthetic wastewater for 60 d (activated sludge was collected with permission from the Public Utilities Board, Singapore). No process data or community data was collected for the sludge before or during the acclimation stage.

After acclimation, the sludge was transferred into the MBR system, complete with hollow fibre membranes, representing the start point for each set of experiments. The MBR system was controlled by a computer with SCADA software (IFIX). The sludge recycle rate was 1.2 L/h. The flux was maintained at 13–17 Liters/m^2^ h (LMH) for all three experiments. The hydraulic and sludge retention times for the three experiments were maintained at 10 h and 25 d, respectively. The parameters, such as membrane flux, TMP, pH, dissolved oxygen, temperature were monitored and automatically recorded. The TOC of the influent and permeate was measured using a multi N/C^®^ 2100s (AnalytikJena). All experiments were operated at room temperature, 25–26°C. Samples were collected at four different TMP ranges, 3–7 kPa, 7–10 kPa, 10–30 kPa and 30–90 kPa, based on previous data suggesting that these were associated with stable operation, the transition towards accelerated TMP rise, during the rapid TMP increase as well as at the maximal pressure.

### Visualisation of biofilms growing on membranes

The HF membranes were cut into 2 cm pieces, immersed in 20 μM SYTO 63 staining solution (Molecular Probes, Invitrogen), incubated at 30°C for 30 min in the dark and then rinsed in phosphate buffered saline (PBS, pH 7.2) for 5 min twice to remove the excess dye. Biofilms on the membranes were then imaged using an inverted confocal laser scanning microscope (LSM 710, Carl Zeiss) (excitation at 633 nm and emission at 650–700 nm). Three dimensional (3D) biofilm images were collected using the “Z stacks” mode and reconstructed using ZEN-2009 light edition (Carl Zeiss) by the “3D” process. The 3D images were converted to the integrated single 2D images through the process of “maximum intensity projection (MIP)”. The surface coverage of the biofilm components on the membrane was calculated using the MIP 2D images by Image J (version 1.46). Images were converted to 8 bit gray images through “image-type”, processed into binary images through “process-make binary”, noisy signals were removed through “Filters” process and the surface coverage of the biofilms were calculated using “Analyze particles”.

### DNA extraction

Total DNA was extracted by a modified CTAB-PEG protocol [24,25]. Briefly, for each sample (biofilm and sludge) and for each time point, 3 replicate samples were collected. The samples were separately extracted, the DNA was quantified by Nanodrop and the samples were separately sequenced. Thus, for the community analysis, all data points represent the average of three replicates. For the extraction of sludge DNA, 0.5 g sludge was collected by centrifugation at 17,000 *g* for 5 min and transferred to the lysing tubes, while for the extraction of biofilm DNA, 10 cm HF membrane were cut into small pieces and put into microfuge tubes containing lysing matrix (MP Biomedicals). Subsequently, 0.5 mL of 5% CTAB lysis solution and 0.5 mL phenol/chloroform/isoamyl alcohol (25:24:1) were added to the tubes. The tubes were placed in a Fast-Prep bead beater (FastPrep-24, M.P. Biomedicals) and shaken using speed setting 5.5 for 30 s. After centrifugation at 17,000 *g* for 5 min, the top aqueous layer was purified by the chloroform/isoamyl alcohol (24:1) and mixed with 2 volumes of 30% PEG solution at 4°C overnight to precipitate the DNA, which was resuspended in DNase and RNase free distilled water and stored at -80°C.

### Quantitative PCR

Quantitative PCR (qPCR) was performed in a LightCycler 480II system (Roche Applied Science). Triplicate samples were used to quantify rRNA gene copy number for biofilm and sludge samples using 16S and 18S specific primers and probes (S1 Table). A two-step amplification procedure, annealing and extension, was performed: 95°C for 10 min, 50 cycles of denaturation step at 95°C for 10 sec and simultaneous annealing and extension step at 60°C for 30 sec. The 16S rRNA gene of *Escherichia coli* K12 and 18S rRNA genes of *Saccharomyces cerevisiae* were used as the positive controls and templates of the construction of standard curves for the bacteria and fungi respectively, while the standard curve for the archeal community was generated from an equal mix of 16S rRNA genes amplified from *Methanobacterium formicicum*, *Methanobrevibacter arboriphilicus*, *Methanospirillum hungatei*, *Methanomicrobium mobile*, *Methanosarcina acetivorans*, *Methanosarcina barkeri*, *Methanosarcina mazei* and *Methanosaeta concilli* and cloned into pGEM as described [26].

### DNA sequencing and sequence processing

The DNA was sequenced by pyrosequencing (Research and Testing Laboratory, Texas, US) [27], using the primers Gray28F and Gray519R (S1 Table) for the bacterial community [28,29], ARC787F and ARC1059R for the archaeal communities [26] and funSSUF and funSSUR (S1 Table) for the fungal community [30]. The sequence data was processed using MOTHUR based on the Costello analysis pipeline [31]. The barcodes, primers and sequences that had poor quality, below 25, were removed from the dataset. The chimeric sequences were identified and removed from the datasets using “chimera.slayer”. Finally, the sequences were aligned against the SILVA 16S and 18S rRNA sequence databases for assigned to taxonomic groups against to the SILVA bacterial and eukaryotic taxonomic reference [32]. The criterion for the sequence classification (similarity to the reference sequence) were Species (> 97%), Genus (94%–97%), Family (90%–94%), Order (85%–90%), Class (80%–85%), and Phylum (75%–80%) [11]. Sequences with similarities below these criteria were classified into unidentified groups for each taxonomic rank. To increase the confidence to the classifying data, a bootstrap based calculator was introduced to the sequence classifying, where the bootstrap value was set to 60% [33]. The non-fungal sequences were removed in the sequence processing step using Mothur software. In the sequence processing, the total sequences were classified first against to the Silva eukaryotic reference using the “classify.seqs” command. After that, the sequence data and taxonomy data for the non-fungi were removed by the process of “remove.lineage”. All sequences have been deposited into the Genbank, where the accession numbers of the sequence packets can be found in S2 Table.

### OTUs and phylotype based community analyses

Two analysis pipelines, OTU and phylotype based, were performed. For the OTU based analysis, the distance of the sequences was calculated by the command “dist.seqs”, followed by a “cluster” process to assign the sequences to OTUs. In this study, the OTU assignment was performed at cutoff of 0.03, which means the sequence dissimilarity in different OTUs is ≥ 3% and the sequence similarity in one OTU is ≥ 97%. Therefore, the cutoff of 0.03 was used to represent Species in the taxonomic classification [34]. The generation of OTU tables and classification of OTUs were performed by the “make.shared” and “classify. otu”. For the phylotytpe based analysis, the data matrix was generated from the taxonomic file instead of the sequence file. The phylotype table was generated through the processing of “phylotype” and “make.shared”. To estimate the sequencing depth, the rarefaction curves were plotted based on the OTUs or phylotypes acquired and the number of sequences was pooled. The relationship between the OTUs, phylotypes and sequences was calculated by MOTHUR using the command of “rarefaction.single”. Meanwhile, the community coverage of sequencing samples was calculated by MOTHUR using the command of “summary. single” [35].

Phylogenetic clustering trees and Nonmetric Multidimensional Scaling plots (NMDS) were created between the samples based on the Bray-Curtis similarity of compositions of OTUs or phylotypes in the different groups [36]. The similarity and dissimilarity between the samples were calculated by the operation “SIMPER” (PRIMER-E) [37]. The contributions of the OTUs and phylotypes to the similarity and dissimilarity were calculated based on the different abundances of phylotypes between the samples and the effluences of phylotype to the community change [38].

## Results

### MBR operation and biofouling behavior

**Fig 1:**
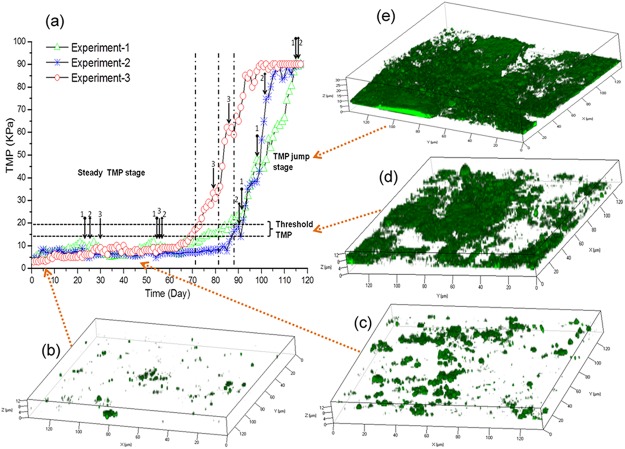
The TMP increase profiles and biofilm growth on membranes during MBR operation. The sampling points for DNA extraction in the TMP increasing process (a) were indicated by the arrows beginning with circles (experiment-1), arrows beginning with diamonds (experiment-2) and normal arrows (experiment 3). The biofilms, represented by the microbial clusters, were stained by SYTO 63 at 3–5 kPa (b), 5–10 kPa (c), 15–20 kPa (d) and 20–80 kPa (e) respectively.

Three replicate MBR experiments were performed to determine the correlation between the microbial community and TMP increase during biofouling. The TOC removal efficiencies for all three experiments were in the range of 95%-98%. Imaging of the biofilm showed that the biomass of the microbial cells increased with time and at 60 kPa, covered approximately 50% of the membrane surface (Fig 1). For each of the three experiments, the TMP profiles were similar over the entire study period. The initial period of operation showed a stable TMP at approximately 3–15 kPa followed by a rapid increase in TMP, called here the TMP jump, where the TMP increased from 20–90 kPa (Fig 1). For all three experiments, the stable operation phase and the TMP jump was separated by an intermediate phase, called here the threshold TMP region, where the TMP was 15–20 kPa. For experiment-1 and 2, which were run at a constant flux of 13–15 LMH, the system required 80–90 d for the TMP to increase from 3 kPa to 15 kPa. For experiment-3, which was run at the constant flux of 15–17 LMH, the TMP rose from 3 kPa to 15 kPa over a 70–75 d period. After the TMP exceeded the threshold 15–20 kPa, the TMP increased exponentially in all three experiments, termed here as the ‘jump stage’ and reached approximately 88–90 kPa, which was the maximum pressure for the MBRs. The TMP jump stage required 24, 20 and 23 d for experiments 1, 2 and 3 respectively. Therefore, in order to determine the relationship between the biofilm associated microbial community and the TMP increase, DNA samples were collected from the activated sludge in the bulk phase and biofilms on the membrane surface in the steady, low TMP stage (3–15 kPa), threshold TMP region (15–20 kPa) and the TMP jump stage (20–90 kPa) to characterize the microbial communities.

### Relative abundance of bacteria, fungi and archaea in biofilms and activated sludge

**Fig 2:**
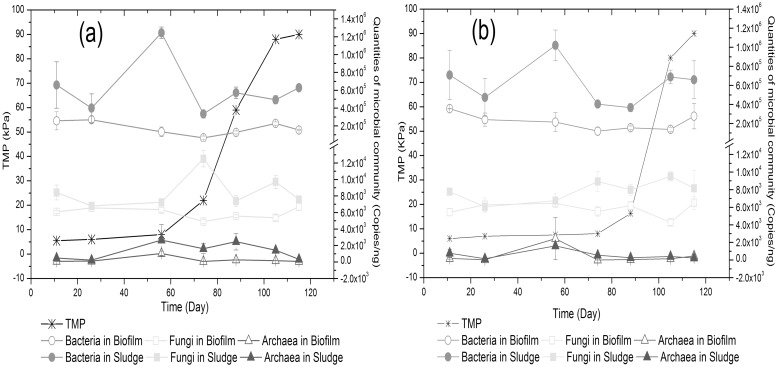
The quantity of microbial populations in biofilm and activated sludge during the MBR operation as determined by quantitative PCR. The left “y” axis shows the TMP at different times. The right “y” axis shows the number of archaea, bacteria and fungi in experiment-1 (a) and experiment-2 (b) respectively as determined by qPCR quantification of rRNA gene copy number. The values are the average number of the triplicate samples. The error bars are the standard errors of the mean (n = 3).

The bacterial, fungal and archaeal communities in biofilm and activated sludge samples were quantified by qPCR. In experiments 1 and 2, the rRNA gene copy number for bacteria ranged between 0.7 × 10^5^–3.5 × 10^5^ copies/ng DNA for biofilms and 3.3 × 10^5^–1.2 × 10^6^ copies/ng for sludge across the entire experiment (Fig 2). The fungal community was present at 4.2 × 10^3^–6.7 × 10^3^ copies/ng DNA and 6.1 × 10^3^–1.2 × 10^4^ copies/ng DNA in the biofilm and sludge samples, respectively (Fig 2). The archaeal community was also detected in the MBR system and was generally similar to the counts observed for the fungi or were one order of magnitude lower in the biofilms (0.2 × 10^2^–2.4× 10^3^ copies/ng DNA) and sludge (1.6 × 10^2^–2.7 × 10^3^ copies/ng DNA). Thus, the bacteria represented between 92.9–98.4% and 95.9–99.2% of the biofilm and sludge communities at all time points (S2 Fig). In addition, the fungi accounted for higher proportions in biofilms (2.3–6.9% in experiment-1 and 1.5–4.4% in experiment-2) relative to in the sludge (0.6–3.6% in experiment-1 and 0.7–2.2% in experiment-2) (S2 Fig). Given that the archaea represented such a small proportion of the community (0.03–0.07%), they were not included in subsequent analyses.

### Bacterial community corresponding to the biofouling process

After removing sequences of poor quality, experiments 1–3 respectively had 224,616 sequences for 24 samples, 265,869 sequences for 24 samples and 92,413 sequences for 18 samples. The average number of sequence reads were 9,359 per sample (experiment-1), 11,077 per sample (experiment-2) and 5,134 per sample (experiment-3). For the bacteria, a total of 11509, 8279 and 4383 OTUs were generated for experiments 1–3 respectively (using a 0.03 cutoff). The rarefaction curves and “summary” analysis indicated that this represented 70–80% of the OTUs for each group of samples in the three experiments (S3 Fig). The bacterial communities were dominated by 1000 OTUs, which accounted for approximately 95% of the total OTUs in each sample (S4 Fig). This provided a high confidence on the sequencing depth and the sampling size of this study and indicated that the data offers an accurate reflection of the bacterial community in the biofouling process of MBRs.

### Compositions of biofilm bacterial community on membrane

#### Dominant bacteria in biofilms at low TMP

It was observed that many OTUs, including the dominant and rare OTUs, were classified into the same bacterial groups based on the taxonomic phylotypes. For example, OTUs 84, 36, 37 and 12 in experiment-1 were all classified to the Order of Burkholderiales, although they matched representatives of the genera *Ralstonia*, *Cupriavidus*, *Pelomonas* and *Ideonella* respectively. Moreover, OTUs 2, 5 and 22 in experiment-1 all were identified as different members of the Genus *Zoogloea* (S3 Table). It was further noted that some of the dominant OTUs were unclassified or uncultured at the taxonomic ranks of Family and Genus but could be differentiated clearly at the Order level (S3 Table). Therefore, the bacterial compositions of the early biofilms were further examined by the phylotypes at the Order level for all three experiments.

The early stages of MBR operation indicated that the biofilm was dominated (at least 0.5% of the relative abundance) by 33 OTUs in both experiments-1 and 2 and 23 OTUs in experiment-3 (S3 Table). These OTUs were classified into 12 Orders (e.g. Actinomycetales, Burkholderiales, Opitutales, Pseudomonadales, Rhodocyclales and Sphingobacteriales etc) based on the Silva bacterial reference with the bootstrap value of 60%. The most abundant OTU in experiment-1 (4.09%) was OTU 7 (Sphingobacteriales; Cytophagaceae; *Flexibacter*), while in experiment-2, the most abundant OTU (4.13%) was OTU 17 (Opitutales; Opitutaceae; *Opitutus*), although these two experiments were operated in parallel. In experiment-3, OTU-3 (Sphingobacteriales; Chitinophagaceae; uncultured) was the most abundant (1.98%).

**Fig 3:**
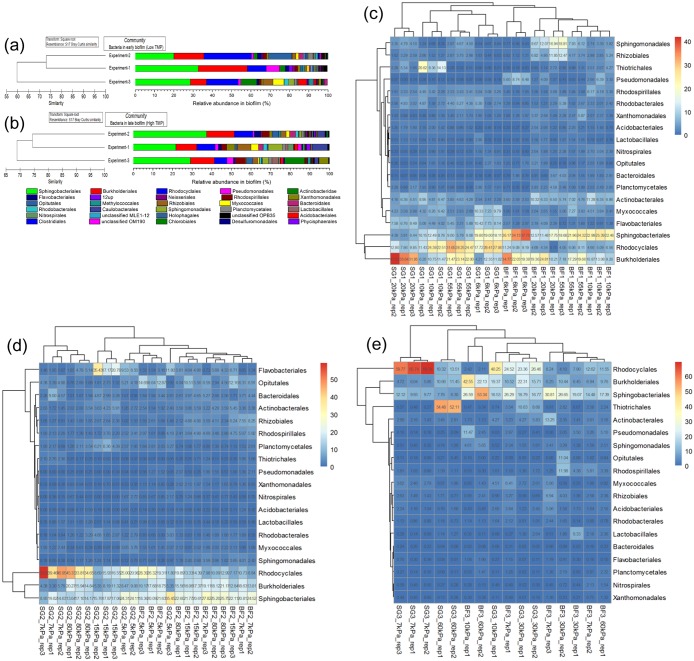
The dominant bacterial communities in biofilms on the hollow fibre membranes. Panels (a) and (b) show the average abundance of bacteria at low (5–7 kPa) and high (30–90 kPa) TMP and the Bray-Curtis similarities of the bacterial communities between the triplicate experiments. Panels (c), (d) and (e) show heatmaps of the key bacteria and their abundances at different TMP in experiments 1, 2 and 3 respectively. In (c), (d) and (e), the “BF” and “SG” in the labels indicate the biofilm and sludge in the experiments. The “rep” in the labels indicates replicate samples. The names on the right show the bacteria at the Order rank. The label in the top right corner shows the relative abundance of certain bacteria in the sludge or biofilm community (%).

When examined at Order level, the biofilms formed at the low TMP (5–7 kPa) shared 59.75% similarity across the three experiments (Fig 3). The biofilm communities in experiments 1 and 2 were more closely related to each other (73.04% in similarity) than the biofilms characterized in experiment-3 at the low TMP stage. Five Orders of bacteria were the dominant groups in all three experiments (between 62–73% of the total community), including Sphingobacteriales, Rhodocyclales, Burkholderiales, Actinomycetales and Flavobacteriales.

#### Dominant bacteria in biofilms at high TMP

The bacterial communities observed at high TMP were dominated by 22, 20 and 17 OTUs at cutoff of 0.03 in experiments 1, 2 and 3, respectively (S4 Table). These OTUs were classified into 13 Orders (e.g. Burkholderiales, Lactobacillales, Rhodocyclales, Sphingobacteriales and Sphingomonadales). At the Order level, the bacterial communities showed 67.47% similarity in the biofilms at high TMP for the 3 experiments (Fig 3). Five Orders were dominant in all three experiments, including Sphingobacteriales, Burkholderiales, Rhodocyclales, Rhodospirillales and Rhizobiales, comprising 55.27%, 51.28% and 67.25% of the bacterial communities, respectively, for the three experiments. The bacterial biofilm communities at high TMP (30–90 kPa) in experiments 1 and 2 had a higher similarity (74.44% similarity) to each other than with the late biofilm community in experiment-3 (67.47% similarity) (Fig 3), which was consistent with the observations from the early biofilms at low TMP.

#### Relationship of bacterial communities in biofilms and activated sludge as the TMP increased

**Fig 4:**
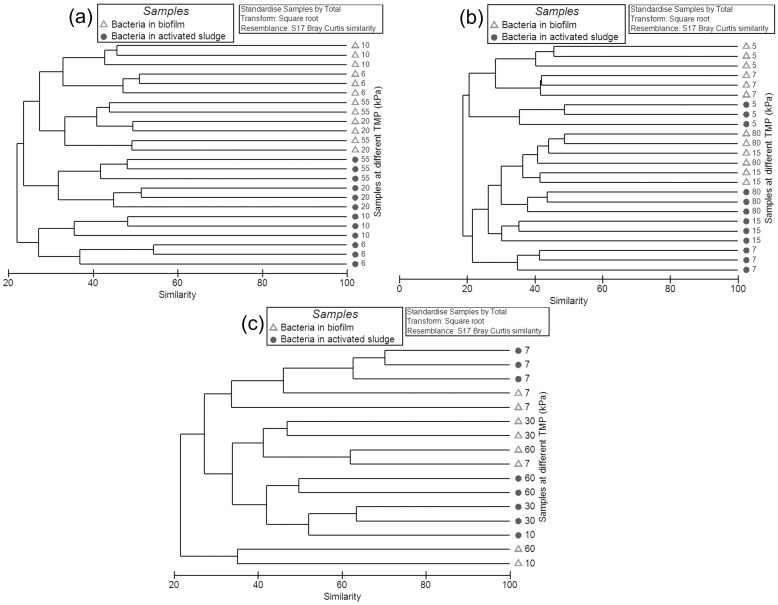
Clustering dendogram of bacterial communities based on the OTUs for biofilms and activated sludge samples found in MBRs for experiment-1 (a), experiment-2 (b) and experiment-3 (c). The circles represent the bacterial communities in the sludge, and the empty triangles represent the bacterial communities in the biofilms. The data for all the samples was the OTUs table at cutoff of 0.03. The numbers in the plots represent the TMP values (kPa) when the samples were collected. The relationships amongst samples were displayed based on the Bray-Curtis similarity between bacterial communities. The values for all samples were generated from the square root transformed data.

**Fig 5:**
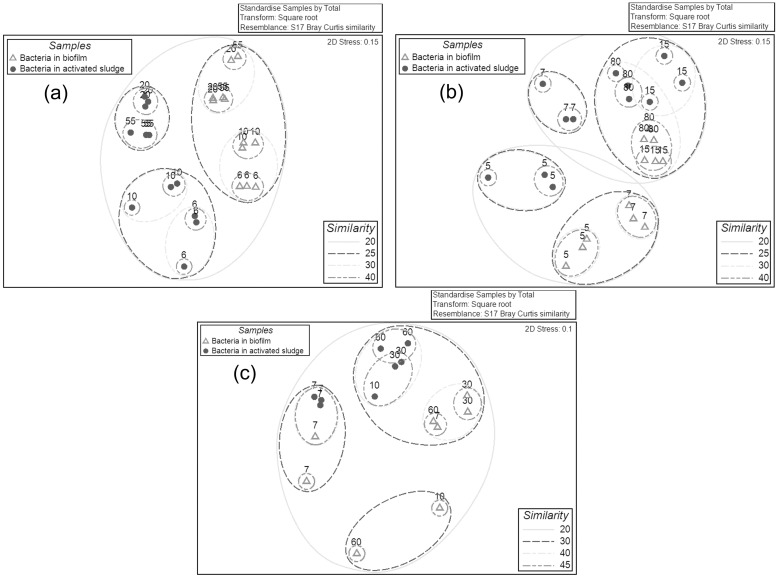
Two dimensional NMDS plots of bacterial communities based on the OTUs for biofilms and activated sludge samples for MBR experiment-1 (a), experiment-2 (b) and experiment-3 (c). The circles represent the bacterial communities in the sludge, and the empty triangles represent the bacterial communities in the biofilms. The data for all the samples was the OTUs table at cutoff of 0.03. The numbers in the plots represent the TMP values (kPa) when the samples were collected. The relationships amongst samples were displayed based on the Bray-Curtis similarity between bacterial communities. The values for all samples were generated from the square root transformed data.

The relationships between bacterial communities at different TMP values were analyzed based on Bray-Curtis similarities based on the bacterial OTU composition. To facilitate the analysis, the clustering dendrogram and NMDS plots were constructed. The bacterial biofilm communities were generally distinct from the activated sludge communities according to the analysis of the clustering dendrogram (Fig 4) and NMDS plots (Fig 5 and S5 Fig). Based on the OTUs at a cutoff of 0.03, in all three experiments, the dissimilarity of bacterial communities between biofilm and sludge ranged from 60–77% at both low (3–15 kPa) and high TMP (15–90 kPa) (S6 Fig). This was apparent for the bacterial community in experiment-1 where the biofilm samples formed separate, distinct branches relative to the sludge based on the NMDS plot (Fig 5). Similar phenomena were also observed for the bacterial communities between the biofilms and sludge in experiments 2 and 3 (Fig 5 and S5 Fig). The same trend was also observed for the phylotype based NMDS analysis (S7 Fig). These data suggest that only a restricted subset of the sludge bacterial community formed biofilms on the membrane.

The biofilm communities at the low and high TMP stages were also distinct from each other. For example, the bacterial communities (experiment 1) formed distinct clusters at 6, 10, 20 and 55 kPa as visualised by the NMDS plot (Fig 5). Similar phenomena were observed in experiments 2 and 3 (Fig 5). This suggests that the membrane biofilm community changed as the pressure increased from low to high TMP. Further, it was found that the majority of the community change in the biofilm occurred before the TMP entered the jump stage (20–90 kPa). For example, in experiment-1, the biofilm bacterial communities at 20 kPa clustered into one group with the communities at 55 kPa (33.5% similarity) relative to the bacterial communities at 6 kPa and 10 kPa (27.5% similarity), which clustered into a separate group. In experiment-2, the biofilm bacterial communities at 15 kPa had a higher similarity to the communities at 80 kPa (37.3% similarity) in comparison with the communities at 5 and 7 kPa (24.7% similarity). Similar phenomena were also observed in the phylotype based analysis (S7 Fig). This showed the biofilm bacterial community at the threshold TMP (15–20 kPa) more closely resembled the biofilms at high TMP (20–90 kPa) than the biofilms at low TMP (3–15 kPa).

#### Comparison of microbial community between the biofilms and sludge

**Fig 6:**
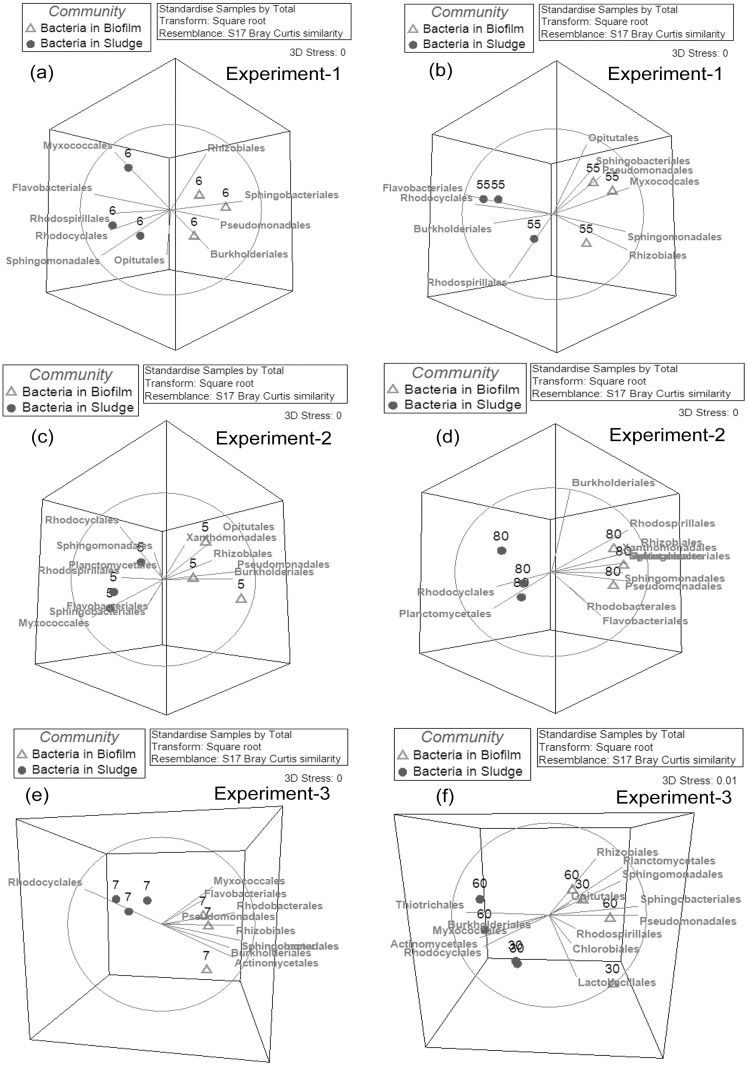
The differentiation of bacterial phylotype compositions between the activated sludge and biofilm in MBRs at 5–7 kPa (a, c, e) and 30–90 kPa (b, d, f). The numbers in the plots represent the TMP values (kPa) when the samples were collected. The branches point to the major bacterial phylotypes that contributed to the community differentiation. The same direction of the samples and branches means the branch-pointed bacteria has higher abundance in the samples.

The above results showed that a subset of bacteria in sludge may be responsible for biofilm formation on the membranes. Thus, the bacterial communities were compared between the sludge and biofilms at the both low and high TMP. The Orders of Burkholderiales, Pseudomonadales and Rhizobiales were enriched in the early biofilms at low TMP (5–7 kPa) in experiments 1–3, respectively, relative to the sludge community (Fig 6). Interestingly, for all three experiments, the Order of Pseudomonadales were present in the sludge at very low abundance (0.14% in experiment-1, 0.25% in experiment-2, 0.05% in experiment-3), but were some of the most dominant organisms in the early biofilms (1.46% in experiment-1, 6.94% in experiment-2, 1.12% in experiment-3). When the TMP increased (20–80 kPa), five Orders of bacteria, including Sphingobacteriales, Pseudomonadales, Sphingomonadales, Opitutales and Rhizobiales, were present at higher abundances in all three experiments (Fig 6). Among those dominant orders, the Sphingobacteriales were a major bacterial group in both the biofilm (17.8–29.1%) and sludge (6.4–14.8%) at high TMP. The other four Orders of bacteria were also highly abundant in the late stage biofilms, but were quite rare in the sludge at the same time point. For example, the Pseudomonadales accounted for 1.3–3.3% of the last stage biofilm community but represented of 0.12–0.25% of the sludge community. In total, these 5 Orders of bacteria accounted for 34.5–41.3% of the biofilm community and 13.9–19.4% of the sludge community at high TMP. This may suggest that there was a strong selection for the sludge bacterial community during the establishment of the early biofilm and late biofilm and that biofilm formation favoured their proliferation.

#### Comparisons of bacterial community in biofilms as the TMP increased

It was noted above that not only were the biofilm communities distinct compared to the sludge community, but also that the biofilm communities also fell into distinct clusters, depending on the specific TMP stage at which they were collected (Fig 6 and S7 Fig). This suggests that the biofilm communities changed during operation of the MBR. The following sections show in detail how those biofilm communities changed over time, with respect to either the increase or decrease in abundance of specific organisms.

#### Increased abundance as the TMP increased over time

**Fig 7:**
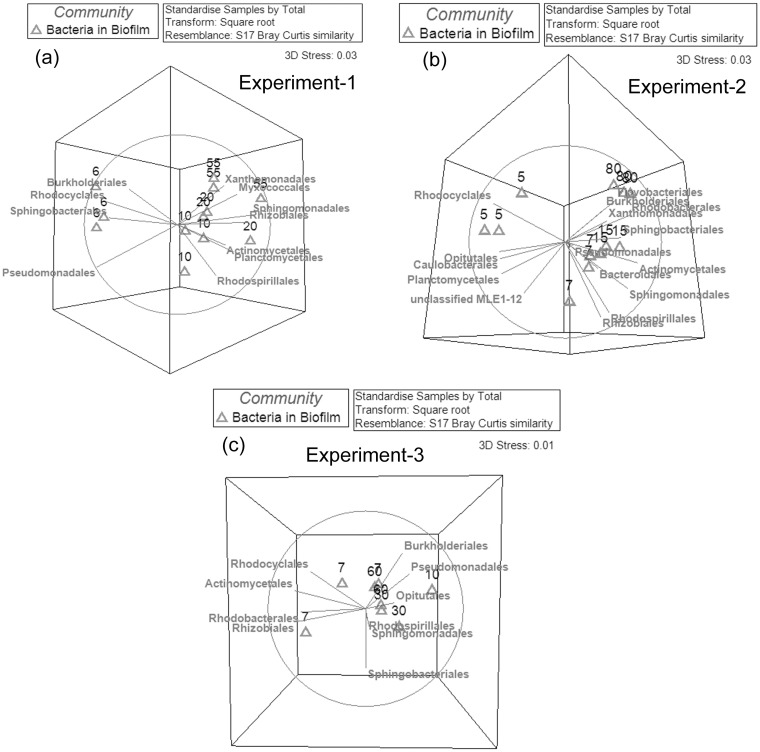
Succession of the bacterial community in the biofilms across the increasing TMP profile. The numbers in the plots represent the TMP values (kPa) when the samples were collected. The branches indicate the major bacterial phylotypes that contributed to the community differentiation. The same direction of the samples and branches means the branch-pointed bacteria has higher abundance in the samples.

Members of the bacterial Orders of Rhodospirillales and Sphingomonadales were observed to increase in abundance in biofilms as the TMP increased in all three experiments (Fig 7). For the bacterial community, the Rhodospirillales accounted for only 1.37%, 1.75% and 1.18% of the bacterial communities at the low TMP stage biofilms for experiments 1, 2 and 3, respectively. In contrast, at the high TMP stage, the abundance of Rhodospirillales increased to 2.64%, 3.63% and 5.01% for experiments 1, 2 and 3, respectively. Similarly, the abundance of Sphingomonadales was 0.55%, 0.56% and 2.88% in the low TMP biofilms but increased to 7.24%, 1.36% and 3.21% in the high TMP biofilms for experiments 1, 2 and 3 respectively.

Additional Orders were found to increase in biofilm abundance across the TMP profile in two of the three experiments. These organisms included Rhizobiales, Actinomycetales, Bacteroidales and Rhodobacterales. The Rhizobiales and Actinomycetales were already the dominant biofilm bacteria at low TMP (1–2% in abundance for Rhizobiales and 2–3% in abundance for Actinomycetales) and became more dominant in biofilms at high TMP (2.7–7% in abundance for Rhizobiales and 3.7–7.8% in abundance for Actinomycetales). For Rhodobacterales, they were the rare bacteria in biofilms at low TMP, where the abundance was 0.89% in experiment-1 and 0.78% in experiment-2. However, when the TMP increased to 55–80 kPa, the abundance of Rhodobacterales increased to 2.01% and 1.44% in experiments 1 and 2, respectively.

#### Decreased abundance as the TMP increased over time

Bacterial of the order Rhodocyclales decreased in abundance in the biofilms as the TMP increased (Fig 7). In the biofilms at the low TMP stage, the Rhodocyclales was present at 9.84% (exp-1) and 24.67% (exp-2) and 16.38% (exp-3) in the bacterial communities, while in the high TMP biofilms, its abundance decreased to 9.3% (exp-1), 9.45% (exp-2) and 6.42% (exp-3). Despite the decreased abundance, these were still the dominant microorganisms in biofilms at high TMP. This could suggest a role for them in establishment of biofilms during the low TMP operation.

#### Co-dominant microbial groups in biofilm at both low and high TMP

Irrespective of whether their relative abundance increased or decreased, some organisms were clearly the dominant organisms in the biofilms across all TMPs. This was particularly obvious for the Sphingobacteriales, Burkholderiales, Rhodocyclales, Actinomycetales and Rhizobiales in bacterial community. Based on their consistent, high abundances, these organisms may be the fundamental organisms responsible for biofilm formation on the MBR membranes.

### Changes in the fungal community during MBR operation

After removing sequences of poor quality, experiments 1–3 respectively had 62,187 sequences for 24 samples, 79,427 sequences for 24 samples and 25,575 sequences for 16 samples. A total of 9,867, 15,191 and 8,255 fungal OTUs (at cutoff of 0.03) were generated for all of the biofilm and sludge samples in experiments 1–3, respectively. The coverage of OTUs indicated by the rarefaction curve and “Summary” analysis was in the range of 80–95% for most of the samples in the 3 experiments (S8 Fig). As for the bacterial data, the fungal communities were also dominated by approximately 1000 OTUs based on the analysis of cumulative dominance (S9 Fig).

### Compositions of fungal community in biofilms at low and high TMP

#### Dominant fungi in biofilms at low TMP

At low TMP, 5–7 kPa, the fungal biofilm community was dominated by 36, 19 or 23 OTUs in experiments 1, 2 or 3 respectively, that each had ≥ 0.5% in total abundance and together accounted for 58.41%, 43.68% and 53.46% of the communities (S5 Table). Most of these OTUs were classified to the Dikarya in the three experiments although no clear identification at the Genus level was possible for most of the OTUs except for OTUs 3 and 9 in experiment-1 and OTUs 5, 19 and 49 in experiment-2, which were all classified as *Candida* sp.

#### Dominant fungi in biofilms at high TMP

When the TMP increased to a high level, the majority of biofilm fungal communities was composed of 23 OTUs in both experiments 1 (55 kPa) and 2 (80 kPa) and 18 OTUs in experiment-3 (60 kPa) and accounted for 53.76%, 47.91% and 23.81% of the fungal communities (S6 Table). As for the low TMP communities, only a few OTUs could be identified to the genus level, i.e. *Candida* sp., such as OTUs 18, 19, 25, 32 and 42 in experiment-1, OTU 39 in experiment-2 and OTUs 81 and 100 in experiment-3, and *Metschnikowia* sp., such as OTU 28 in experiment-1 and OTU 118 in experiment-2. The remainder were either identified at the subkingdom level of Dikarya, or could not be assigned to any taxonomic group.

#### Relationship of fungal communities in biofilm and activated sludge across the increasing TMP

**Fig 8:**
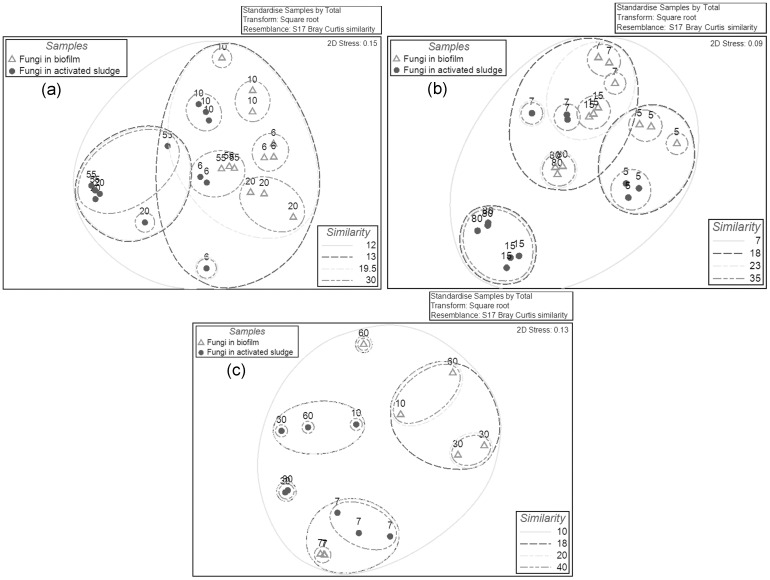
The NMDS plots of fungal communities (two dimensional) based on the OTUs for biofilms and activated sludge samples in the experiment-1 (a), experiment-2 (b) and experiment-3 (c). The circles represent the fungal communities in the sludge, and the empty triangles represent the fungal communities in the biofilms. The data for all the samples was the OTUs of 18S rDNA at cutoff of 0.03. The numbers in the plots represent the TMP values (kPa) when the samples were collected. The relationships amongst samples were displayed based on the Bray-Curtis similarity between fungal communities. The values for all samples were generated from the square root transformed data.

The fungal communities were observed to cluster separately for the activated sludge and biofilm samples based on the clustering dendrogram analysis (S10 Fig) and NMDS plots (Fig 8 and S11 Fig), indicating that members of the fungal community may preferentially form biofilms relative to other community members. The dissimilarity of fungal sludge and biofilm communities ranged from 70–83.2% at 5–10 kPa in all three experiments but increased (90.2–96.6%) when the TMP increased to 15–80 kPa (S12 Fig), which implied a further selection occurred in the fungal community during biofilm formation at high TMP.

## Discussion

Microorganisms have a strong capacity to form surface associated communities encased in a self produced extracellular matrix, called biofilms and have been shown to do so in almost every habitat investigated, including man-made industrial systems. Moreover, these biofilms are communities, comprised of a broad range of different species, and often include members of all three domains of life. These communities gain a range of potential benefits including resource sharing, cross-protection from stress and habitat development. For example, the hydrogen producing bacteria, *Clostridium* sp., co-occurred with the non-hydrogen producing bacteria, *Klebsiella* sp., and *Streptococcus* sp. to form anaerobic hydrogen producing granules, where the *Klebsiella* sp. first consumes the oxygen and the *Streptococcus* sp. secretes high amounts of extracellular polymeric substances to form the interior biofilm structure and anaerobic conditions, followed by growth of *Clostridium* sp. surrounding the *Streptococcus* sp. [39]. Such interactions help to explain why mixed community biofilms are so successful in nature and why they are difficult to control in industrial settings. Membrane bioreactors function by putting hollow fibre membranes into an activated sludge tank, a highly diverse microbial community, and hence it is not surprising that such membranes become fouled by microorganisms forming biofilms on their surfaces, which is one aspect of biological or biofouling. Biofouling is responsible for increased energy costs associated with fouling as well as chemical costs for cleaning as well as replacement of irreversibly fouled membranes. Therefore, there is a strong desire to understand the microbial community and the process of biofilm formation on such membranes to try to identify targeted approaches to reduce or remove fouling organisms.

### Bacteria are the dominant fouling organisms in the MBR system

To first understand which taxa of microorganisms were primarily responsible for biofilm formation on hollow fibre membranes and the reduced system performance, as quantified by the change in TMP, qPCR was performed and demonstrated that bacteria represented the dominant taxa in the biofilms that formed on the MBR membranes relative to fungi and archaea. It has been previously reported that the Proteobacteria and Bacteroidetes were prevalent in biofilms [10,11] and that Bacteroidetes and the Beta-proteobacteria (represented by Burkholderiales) were the co-dominant bacteria in membrane biofilms and in activated sludge [7]. Interestingly, in this study, the Beta-proteobacteria (such as Rhodocyclales and Burkholderiales) and Bacteroidetes (such as Sphingobacteriales and Flavobacteriales) were also the dominant bacteria in the early biofilms at low TMP. As the TMP increased, these same organisms remained dominant and thus, these organisms may be specialized for biofilm formation under the conditions tested here. Thus, these organisms are likely to be responsible for biofouling of the MBR. This contrasts with previous studies indicating that the dominant biofilm forming bacteria were Gammaproteobacteria [11]. The differences in the dominant bacterial community members may be related to experimental differences. For example, in the work of Lim *et al*. (2012), the synthetic wastewater contained glucose at 1,000 mg/L and peptone at 50 mg/L, while the nutrients used in this study contained lower glucose (320 mg/L) and higher peptone (80 mg/L) concentrations. It seems clear that the nutrient contents of the artificial waste-water used here and in other studies will select for different species and that this community composition will differ somewhat from communities cultivated using real waste-water, however, the communities are almost always dominated by proteobacteria. However, the key observation here, that the biofilm community represents a distinct sub-set of the sludge community and that the biofilm community changes with the increase in TMP, may be common to the MBR process. Future work focusing on the change in microbial communities associated with operational MBR plants during different TMP stages or laboratory reactors using real waste-water would be of interest to address this hypothesis.

Although the fungi were also detected on the membrane biofilms (1.5–6.9%), most of the dominant fungal communities were unclassified by 18S rRNA gene sequencing. This may not be surprising as the majority of the current biofilm studies have focused on the role of the bacteria in biofouling process, resulting in the lack of the reference database of the fungal community at the present [15]. Most of the identified fungal OTUs matched *Candida* sp., indicating the *Candida* sp. may play an important role in the construction of fungal biofilm in MBRs, although *Candida* sp. were present at very low abundances, so it seems unlikely that these organisms contribute significantly to biofouling.

### Random attachment or selective attachment of the sludge community during the formation of biofilm

The microorganisms in the activated sludge would logically have to be the source of the communities that eventually form on the membranes and one interesting question is how biofilm formation is initiated and ultimately develops, e.g. is attachment and development stochastic or ordered. In this study, the communities in the sludge and biofilm showed high levels of dissimilarity (S6 and S12 Figs). Furthermore, the biofilm samples formed distinct clusters relative to the sludge based on the NMDS plot, suggesting they were significantly different from the sludge community. These results were similar to the observations published comparing biofilm and activated sludge samples, where it was observed that the bacterial community in the biofilm differed from the community in sludge during the operation of a flat-sheet membrane MBR [40]. Therefore, the colonization of sludge microorganisms onto the membrane may not be random, but rather may occur in a selective fashion during the process of biofilm formation.

Indeed, some microorganisms, such as the Burkholderiales, Pseudomonadales and Rhizobiales, were enriched in both the early biofilms at low TMP and late biofilms at high TMP relative to the sludge community, indicating that these microorganisms had stronger competence in constructing the biofilm rather than living in sludge. Interestingly, while the Pseudomonadales were not in the top five most abundant biofilm bacteria, they were present in the biofilm at higher abundance than in the sludge. This was consistent with the microbial community growing on an air-diffusion biocathode in a microbial fuel cell system where members of the Rhizobiales and Pseudomonadales were present in higher proportions in the biofilms relative to the sludge [41]. Further, the Rhizobiales and Burkholderiales were also the major populations in biofilms formed in drinking water distribution systems [42]. Thus, these bacterial groups may generally be good biofilm forming organisms found in water systems. It was of interest to note that the Rhodocyclales were the dominant microorganisms in both the biofilm and sludge, even though their abundances were lower in biofilms than in sludge. The Rhodocyclales have been reported to be significant components of the sludge community in wastewater treatment systems [43] and were also proposed to be important in the biofouling in MBRs [44] and groundwater treatment facilities [45]. The results here indicated that the Rhodocyclales may be less competitive during biofilm formation on membranes but still played an important role in the construction of biofilm.

### Succession of the biofilm microbial community during the TMP increase

Although the biofilms were composed of similar microbial communities (at the Order level) at low and high TMP, the contributions of the specific microbial groups to the whole biofilm community differed at low and high TMP. For the bacterial community, in comparison with the biofilm at low TMP, the Alpha-proteobacteria (represented here by Rhodospirillales, Sphingomonadales and Rhizobiales) became more dominant in late biofilms at high TMP, while the Rhodocyclales decreased the abundance in late biofilm. This was consistent with the report that the Alpha-proteobacteria increased in abundance in late biofilms in an MBR system [11]. It has been suggested that members of the Sphingomonadales were pioneer bacteria that initiate biofilm formation and flourish during late biofilm formation in microfiltration and ultrafiltration based MBR systems [46,47], due to their swarming and twitching based motility as well as and polysaccharide production [48]. This was consistent with the results presented in this study, which showed that the Sphingomonadales were not highly abundant in the biofilms at low TMP but also became more prevalent in the biofilm at high TMP. These results indicated that the formation of biofilm in the MBR system may involve the succession of microbial groups or species that are best suited for biofilm formation or growth at different stages of MBR operation.

### The succession of the biofilm community may be responsible for the TMP jump

It was particularly interesting to observe that the biofilm communities clustered into different groups, which correlated with the TMP profile at the time of sampling. Coincidently, the TMP increased at different rates for these sampling times, initially showing a steady increase followed by an exponential increase and these two TMP profiles were separated by an intermediary or threshold TMP stage at 15–20 kPa. It is therefore possible that the change in rate of TMP increase may represent a selective pressure that resulted in the observed change in biofilm community structure. It is also possible, that the changing biofilm community altered the water permeability at the membrane surface, and hence resulted in the increasingly rapid TMP increase. While it is not possible to state with certainty, the latter hypothesis is favoured here as the bacterial biofilm community was observed to change prior to the rapid increase in TMP during the jump or exponential phase. One of the key questions that remains to be answered, which would partially address these hypotheses, would be the specific selection pressure that drives the observed changes in community composition. For example, if the local conditions at the membrane surface change, this could select for different organisms. It is not clear that bacteria on the surfaces of the membranes would experience a change in pressure, since the suction is applied from the lumen side of the membrane. However, it has been shown that loss of membrane performance is associated with an increased solute concentration, the concentration polarization effect [49]. This increased solute concentration may thus favour the growth of organsims that tolerate increased salt or osmotic pressure at the membrane surface [50]. Additionally, the concentration polarization effect has been suggested to increase local nutrient concentrations, which could encourage growth of microorganisms as the membrane surface. Alternatively, as noted above, growth of the community at the membrane surface could alter local concentrations of nutrients and oxygen, again, driving selection for organisms that are specialized in growth under such conditions. If this process selects for organisms that secrete increased amounts of EPS, this could subsequently result in blockage of the membrane pores, leading to decreased flux, or increased pressure for systems operated under constant flux. Further work will be required to better test these hypotheses, but if the changing biofilm community drives the increase in TMP, it would argue that strategies should be developed to target those key biofilm forming organisms to control MBR biofouling to improve operational performance and to reduce overall costs.

## Conclusions

The microbial community was compared for both sludge and biofilm samples and the changes in community composition were related to changes in MBR performance, as determined by the change in TMP. The results suggested that the biofilm was initiated from a specific group of bacteria and fungi and that this community changed in composition as the TMP increased. Further, the biofilm community associated with the low pressure operation phase was significantly different from the community associated with the membranes at the time the TMP exceeded the threshold pressure which was followed by an exponential, jump phase where the pressure rapidly reached its maximum for the system. Given that the biofilm initiates from a specific subset of bacteria present in the sludge, it may be possible to target those organisms to ultimately delay their incorporation into the biofilm and hence delay the TMP jump. It will also be particularly interesting to understand the mechanism that results in the subtle community shift that is associated with the TMP jump.

## Supporting information

S1 Fig The MBR systems used in this project. (a) The schematic view of the external submerged MBR. (b) The schematic view of the internal submerged MBR. (c) The configuration of the “curtain” style HF membrane module.
(TIF)

S2 Fig The relative abundance of archaea, bacterial and fungal communities in biofilm (a) and sludge (b) during the MBR operation in experiment-1. (TIF)

S3 Fig The rarefaction curves and coverage of OTUs in experiments 1, 2 and 3 respectively. (TIF)

S4 Fig The cumulative dominance of OTUs in the bacterial community in experiments 1, 2 and 3. (TIF)

S5 Fig Three dimensional NMDS plots of bacterial communities based on the OTUs for biofilms and activated sludge samples in the experiment-1 (a), experiment-2 (b) and experiment-3 (c). The blue circles represent the bacterial communities in the sludge, and the empty triangles represent the bacterial communities in the biofilms. The data for all the samples was the OTUs table at cutoff of 0.03. The numbers in the plots represent the TMP values (kPa) when the samples were collected. The relationships amongst samples were displayed based on the Bray-Curtis similarity between bacterial communities. The values for all samples were square root transformed.
(TIF)

S6 Fig The dissimilarity of bacterial communities between biofilm and sludge at different TMP profiles. The dissimilarity values were calculated from the biofilm and sludge samples at the same TMP through the “SIMPER” process in PRIMER v6.
(TIF)

S7 Fig The 3 dimensional NMDS plots of bacterial communities based on the bacterial phylotype at Order level for biofilms and activated sludge samples in the experiment-1 (a), experiment-2 (b) and experiment-3 (c). The blue circles represent the bacterial communities in the sludge, and the empty triangles represent the bacterial communities in the biofilms. The numbers in the plots represent the TMP values (kPa) when the samples were collected. The relationships amongst samples were displayed based on the Bray-Curtis similarity between bacterial communities. The values for all samples were square root transformed.
(TIF)

S8 Fig The rarefaction curves and coverage of OTUs for the fungal communities in experiments 1, 2 and 3 respectively. (TIF)

S9 Fig The cumulative dominance of OTUs at cutoff of 0.03 for the bacterial communities in experiments 1, 2 and 3. (TIF)

S10 Fig Clustering dendrograms of fungal communities based on the OTUs for biofilms and activated sludge samples in the experiment-1 (a), experiment-2 (b) and experiment-3 (c). The blue circles represent the fungal communities in the sludge, and the empty triangles represent the fungal communities in the biofilms. The data for all the samples was the OTUs of 18S rDNA at cutoff of 0.03. The numbers in the trees represent the TMP values (kPa) when the samples were collected. The relationships amongst samples were displayed based on the Bray-Curtis similarity between fungal communities. The values for all samples were square root transformed.
(TIF)

S11 Fig The NMDS plots of fungal communities (3 dimensional) based on the OTUs for biofilms and activated sludge samples in the experiment-1 (a), experiment-2 (b) and experiment-3 (c). The blue circles represent the fungal communities in the sludge, and the empty triangles represent the fungal communities in the biofilms. The data for all the samples was the OTUs of 18S rDNA at cutoff of 0.03. The numbers in the plots represent the TMP values (kPa) when the samples were collected. The relationships amongst samples were displayed based on the Bray-Curtis similarity between fungal communities. The values for all samples were square root transformed.
(TIF)

S12 Fig The dissimilarity of fungal communities between biofilm and sludge at different TMP. The dissimilarity values were calculated from the biofilm and sludge samples at the same TMP through the “SIMPER” process in PRIMER v6.
(TIF)

S1 Table The primers and probes used in this study. (DOCX)

S2 Table The accession numbers of the sequence packets in Genbank in the 3 experiments. (DOCX)

S3 Table The dominant bacterial OTU s in biofilms at the low TMP in the 3 replicate experiments (a). (DOCX)

S4 Table The dominant bacterial OTUs in biofilms at the high TMP in the 3 replicate experiments. (DOCX)

S5 Table The dominant fungal OTUs in the early biofilms at low TMP in experiment-1, 2 and 3. (DOCX)

S6 Table The dominant fungal OTUs in the late biofilms at high TMP in experiment-1, 2 and 3. (DOCX)
