# Supplementary figures and images for: Succession of biofilm communities responsible for biofouling of membrane bio-reactors (MBRs)
Source: PLoS One. 2017 Jul 7;12(7):e0179855. doi: 10.1371/journal.pone.0179855 (PMC5501448; doi:10.1371/journal.pone.0179855)

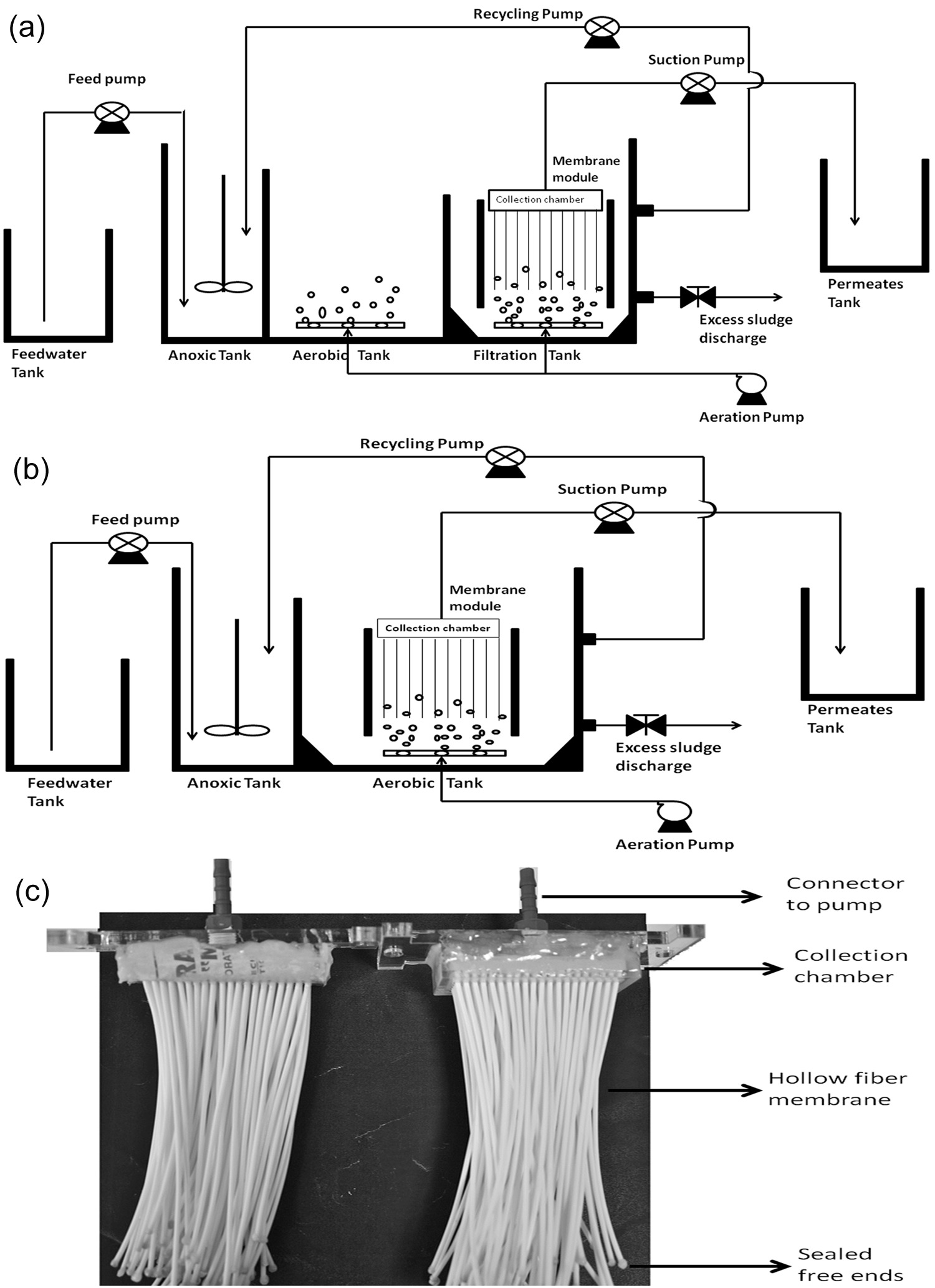

Supplement: S1 Fig — (a) The schematic view of the external submerged MBR. (b) The schematic view of the internal submerged MBR. (c) The configuration of the “curtain” style HF membrane module. (TIF) [file pone.0179855.s001.tif]

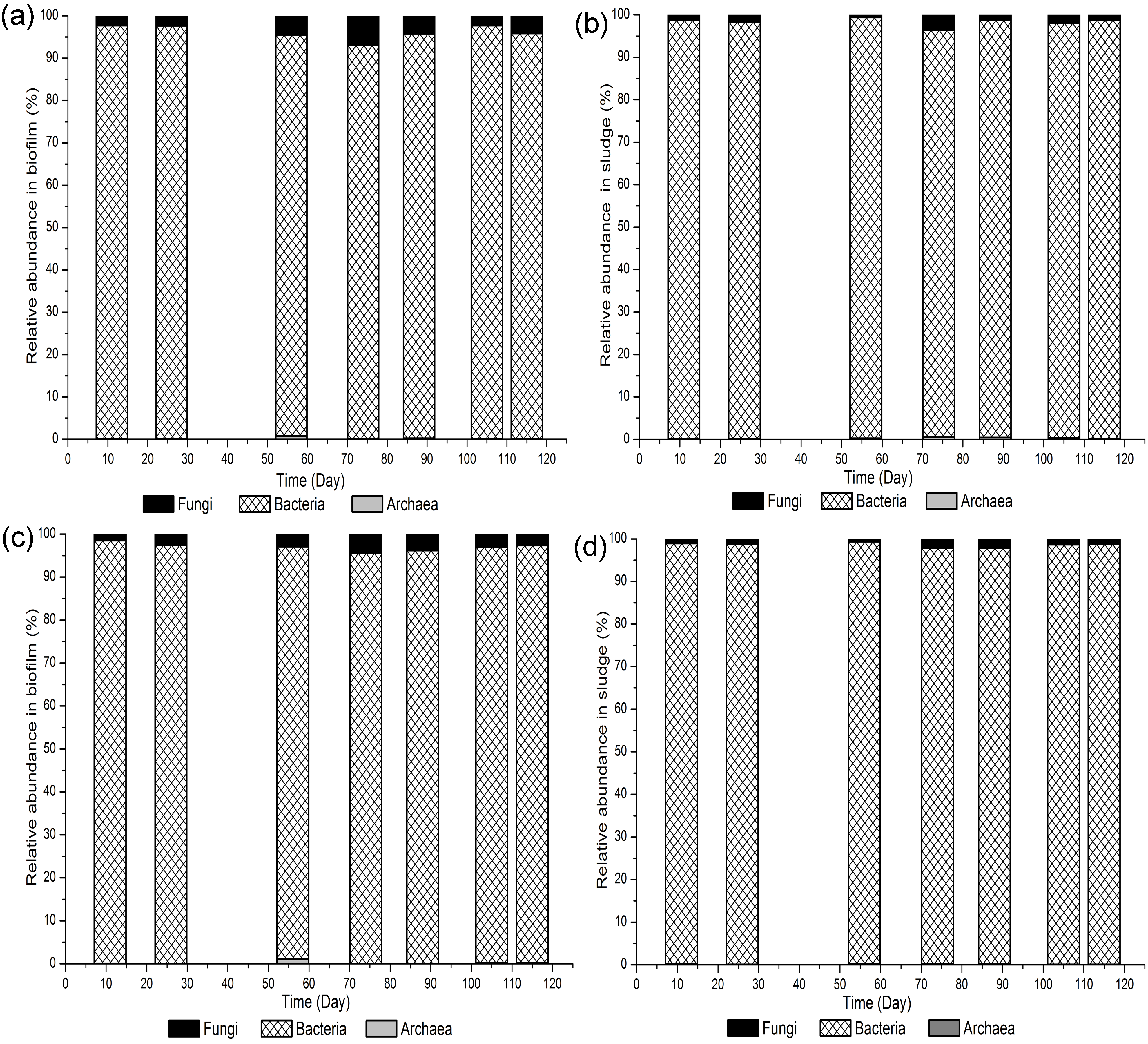

Supplement: S2 Fig — (TIF) [file pone.0179855.s002.tif]

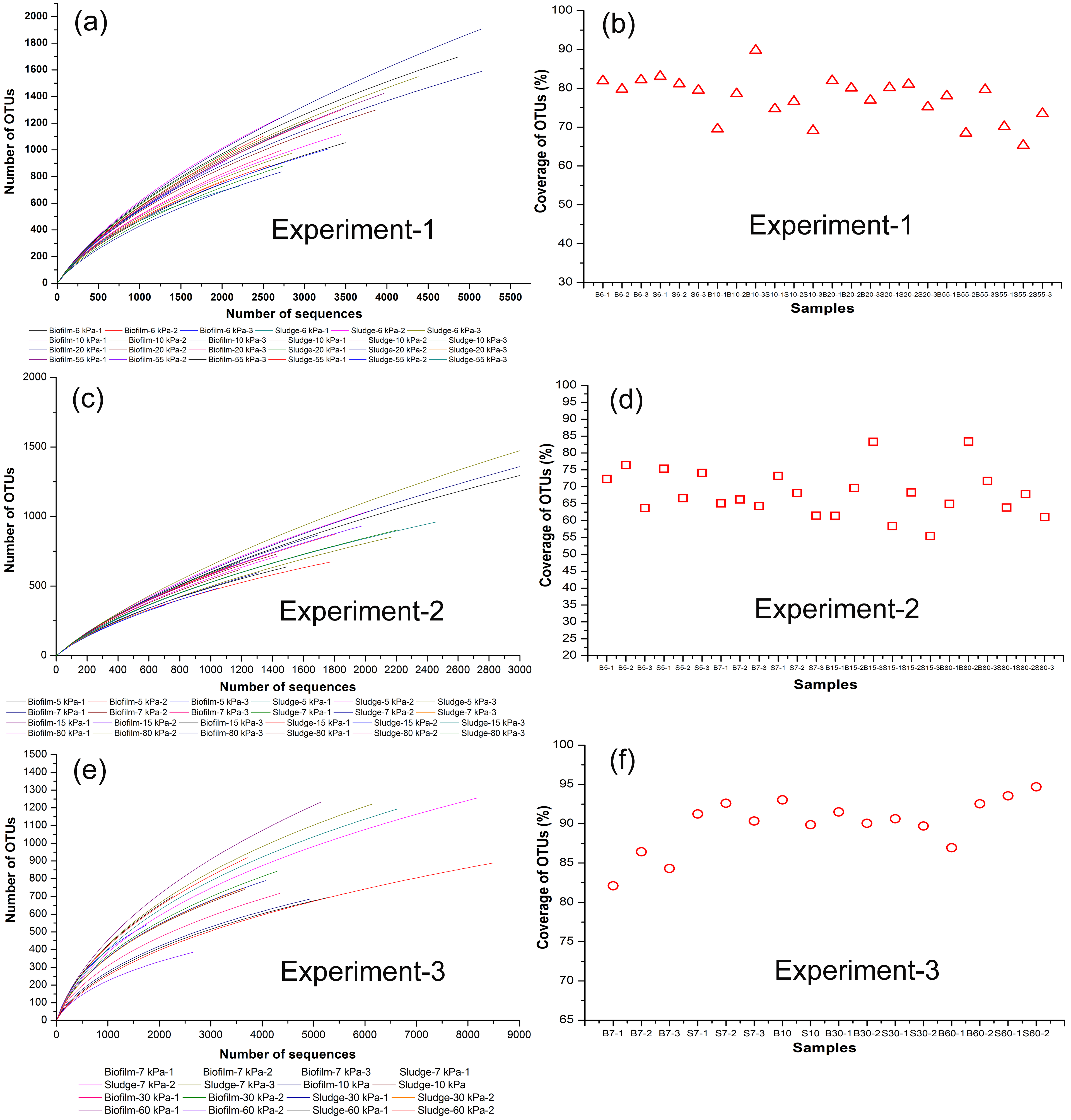

Supplement: S3 Fig — (TIF) [file pone.0179855.s003.tif]

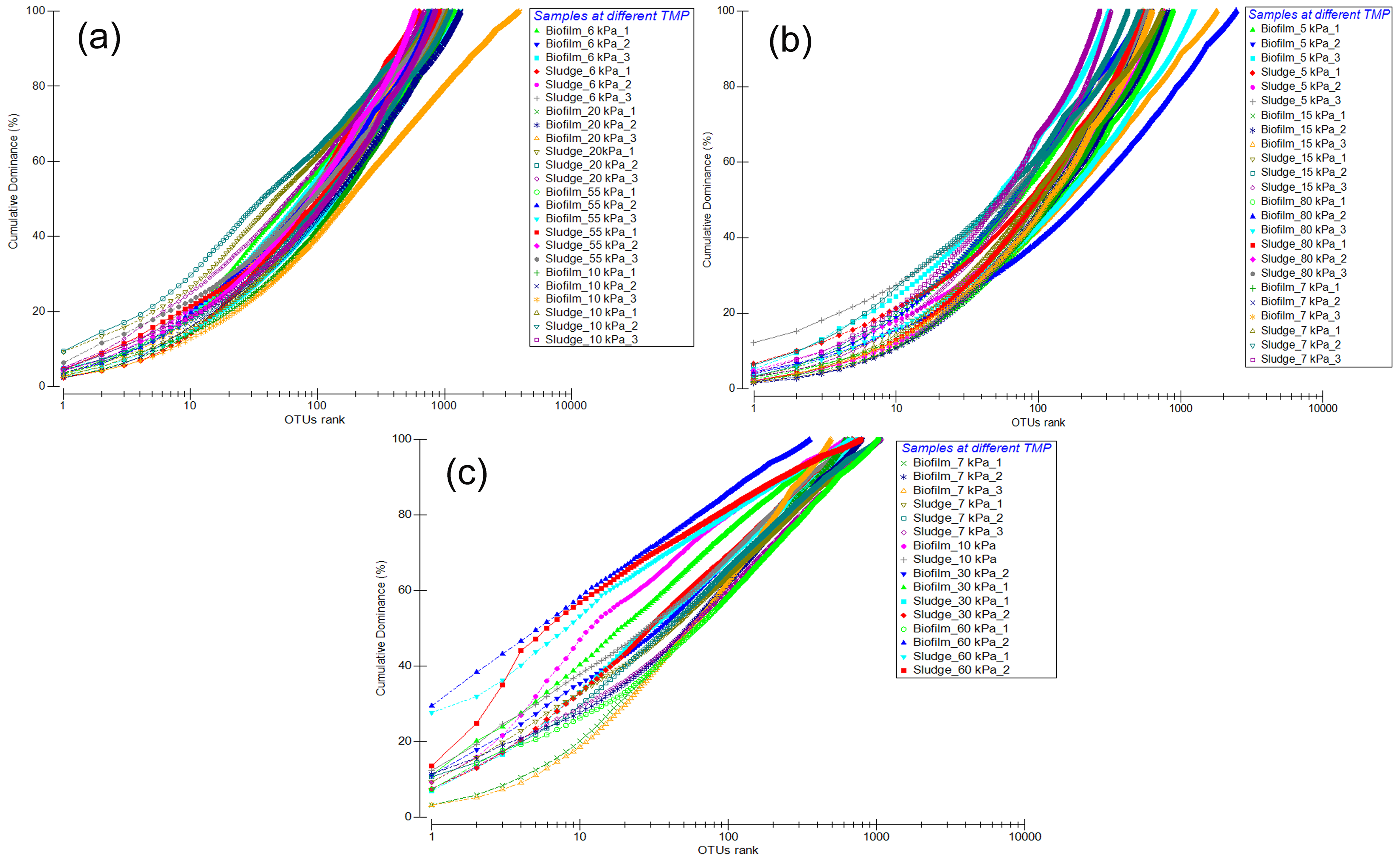

Supplement: S4 Fig — (TIF) [file pone.0179855.s004.tif]

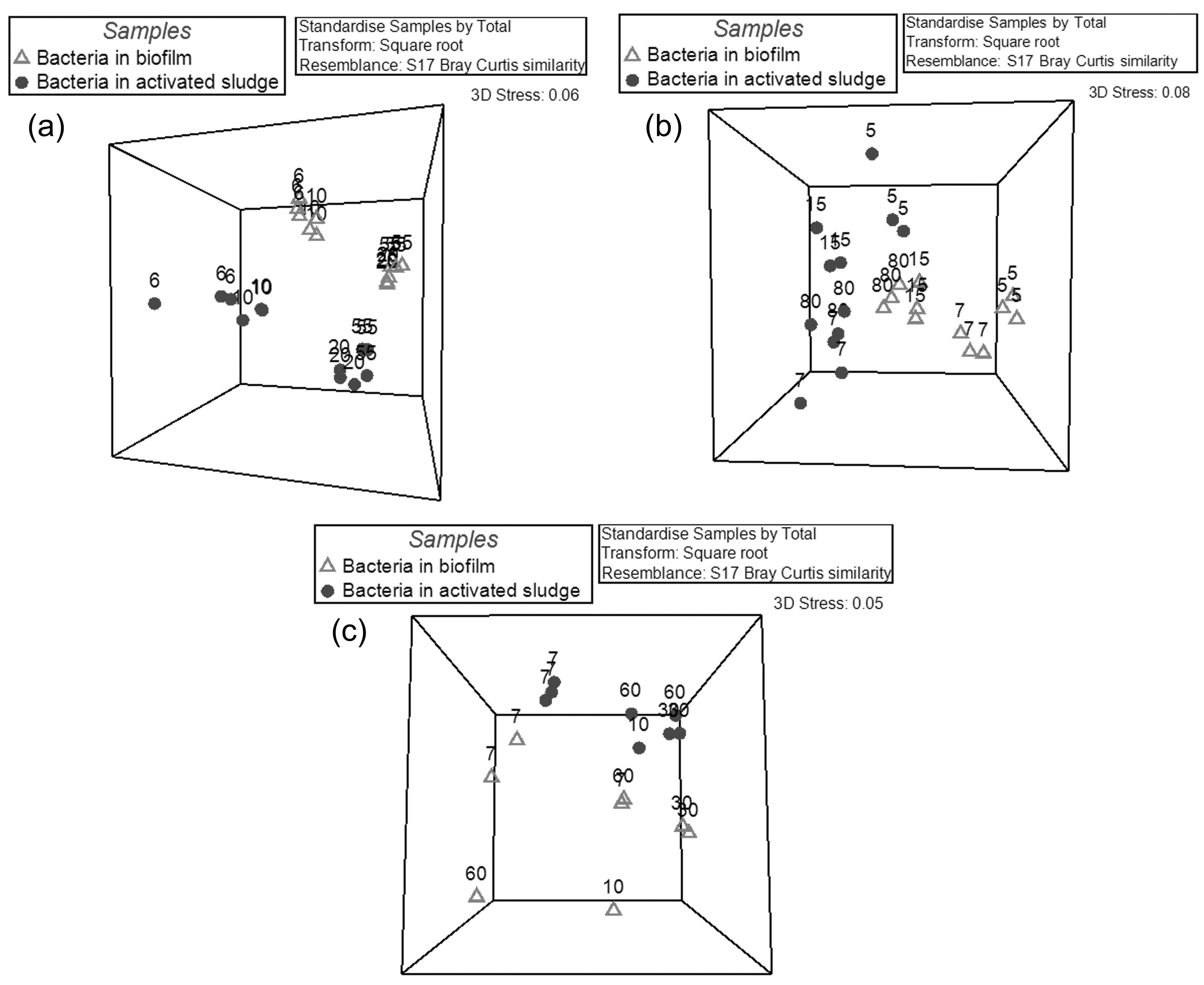

Supplement: S5 Fig — The blue circles represent the bacterial communities in the sludge, and the empty triangles represent the bacterial communities in the biofilms. The data for all the samples was the OTUs table at cutoff of 0.03. The numbers in the plots represent the TMP values (kPa) when the samples were collected. The relationships amongst samples were displayed based on the Bray-Curtis similarity between bacterial communities. The values for all samples were square root transformed. (TIF) [file pone.0179855.s005.tif]

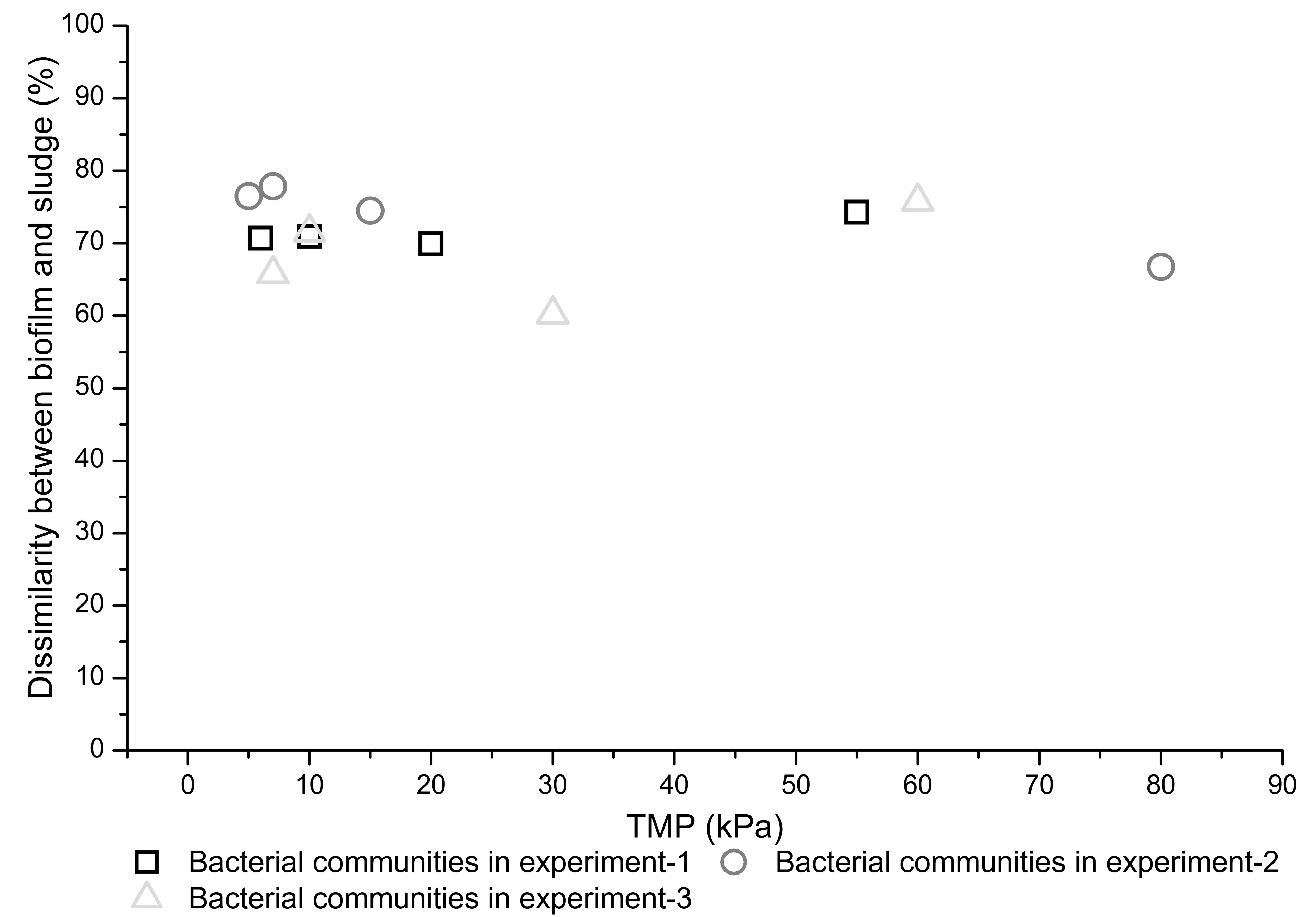

Supplement: S6 Fig — The dissimilarity values were calculated from the biofilm and sludge samples at the same TMP through the “SIMPER” process in PRIMER v6. (TIF) [file pone.0179855.s006.tif]

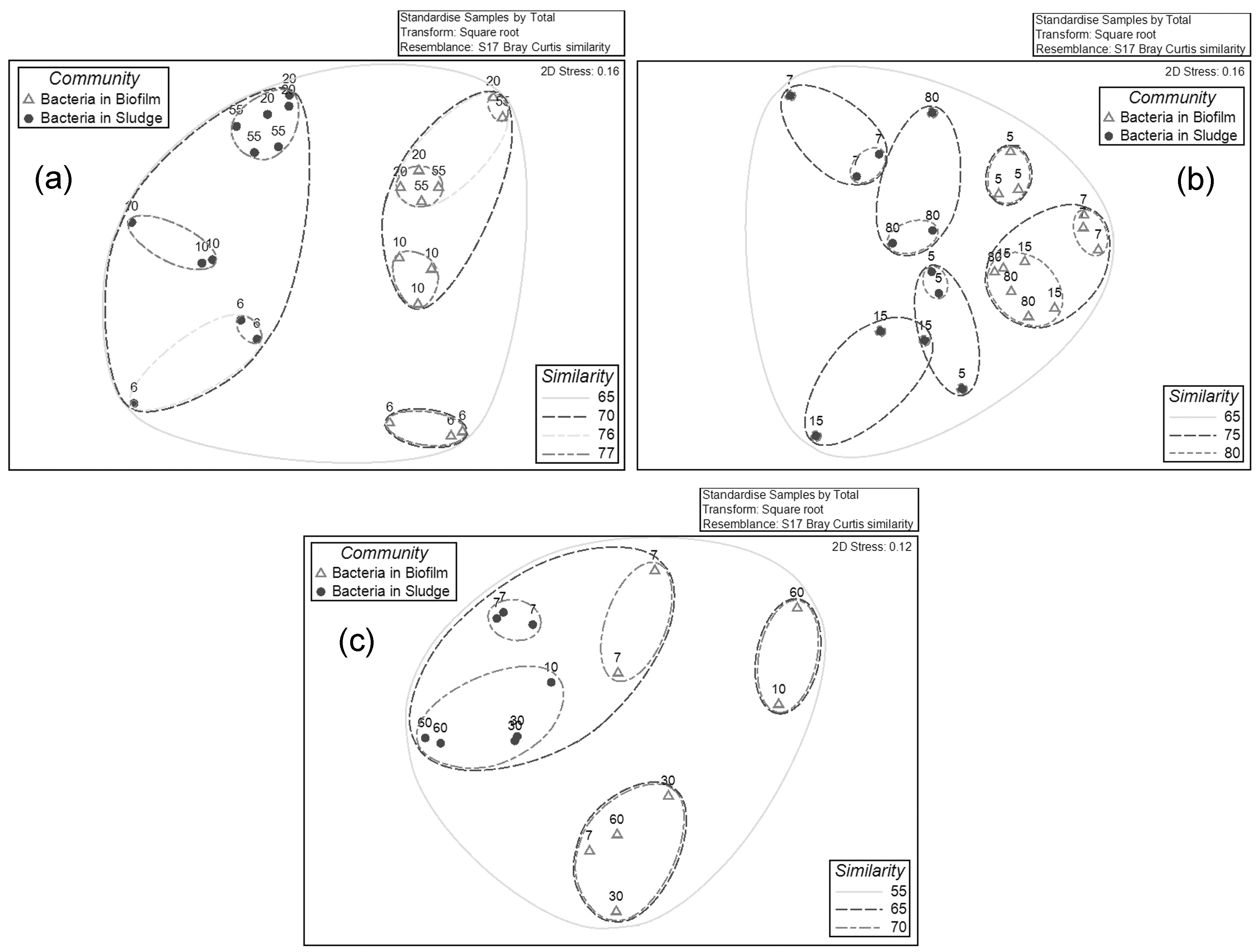

Supplement: S7 Fig — The blue circles represent the bacterial communities in the sludge, and the empty triangles represent the bacterial communities in the biofilms. The numbers in the plots represent the TMP values (kPa) when the samples were collected. The relationships amongst samples were displayed based on the Bray-Curtis similarity between bacterial communities. The values for all samples were square root transformed. (TIF) [file pone.0179855.s007.tif]

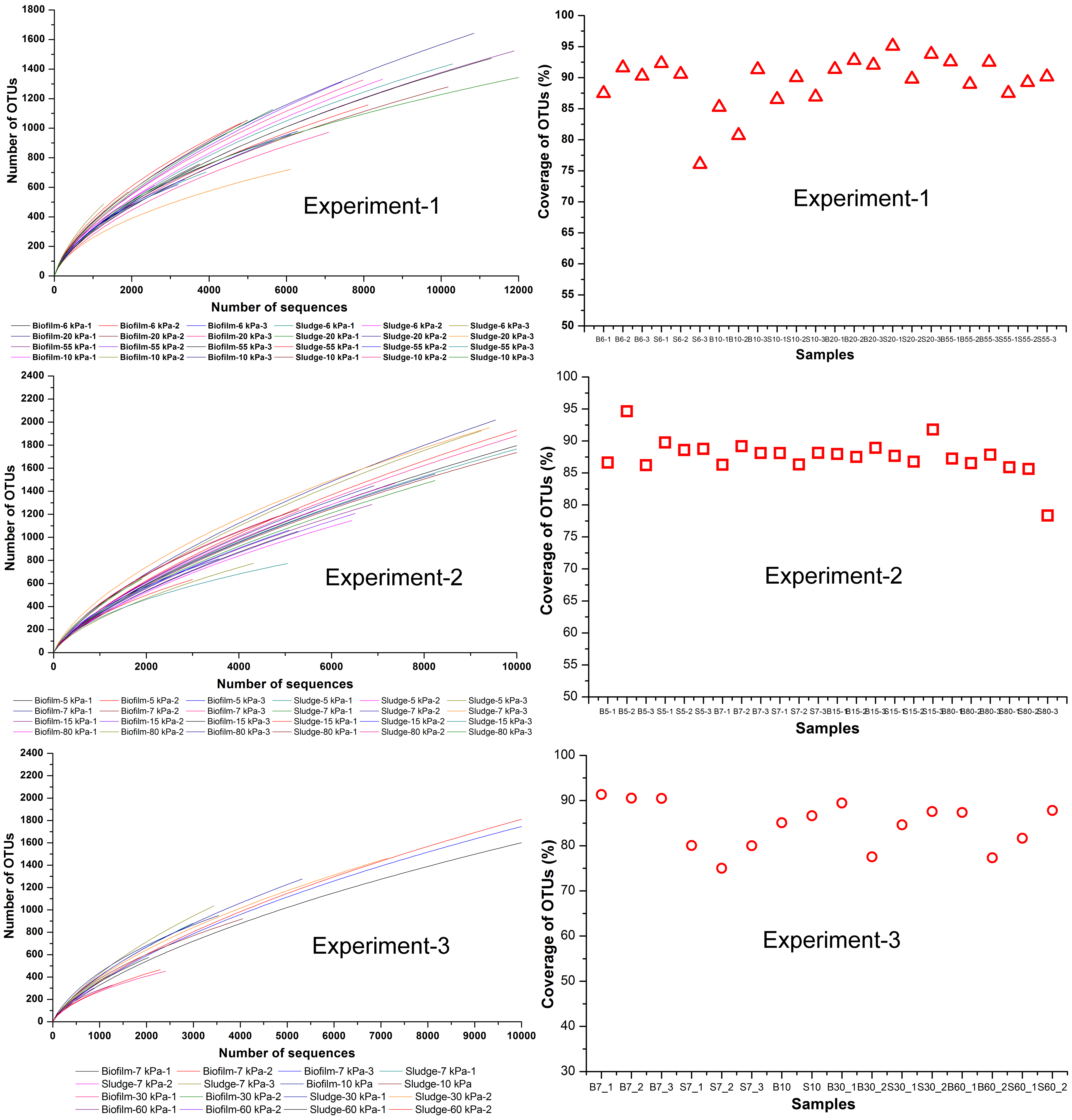

Supplement: S8 Fig — (TIF) [file pone.0179855.s008.tif]

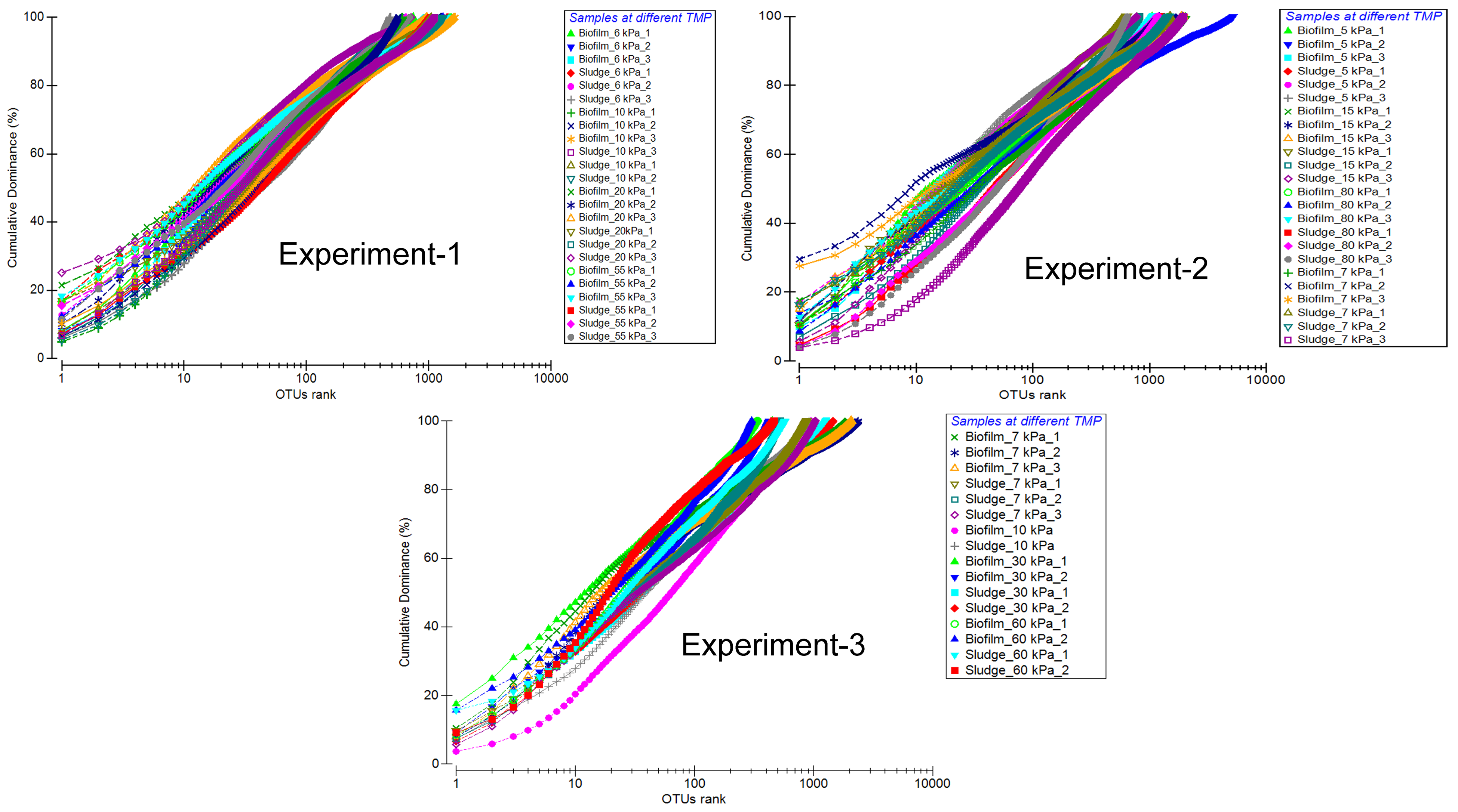

Supplement: S9 Fig — (TIF) [file pone.0179855.s009.tif]

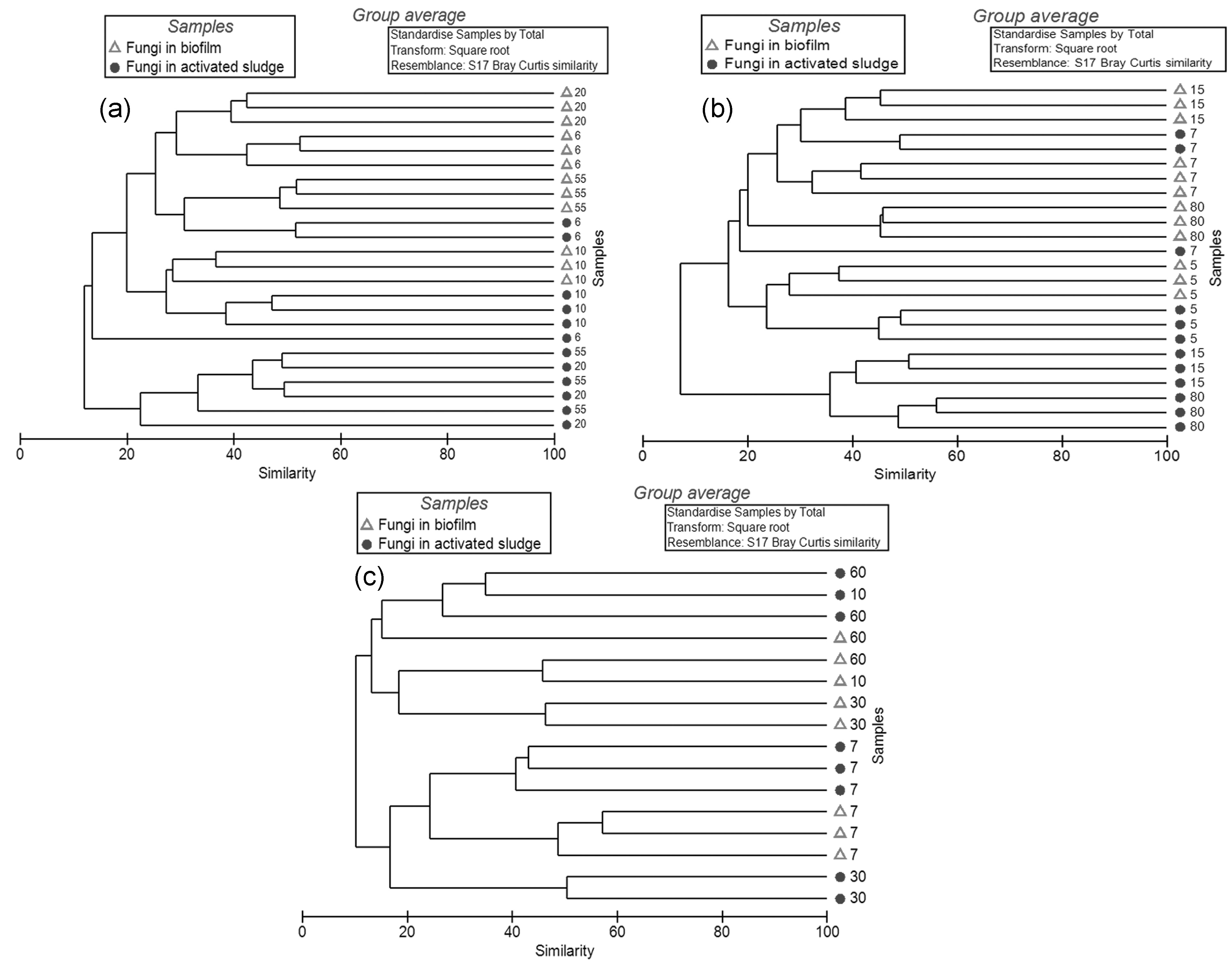

Supplement: S10 Fig — The blue circles represent the fungal communities in the sludge, and the empty triangles represent the fungal communities in the biofilms. The data for all the samples was the OTUs of 18S rDNA at cutoff of 0.03. The numbers in the trees represent the TMP values (kPa) when the samples were collected. The relationships amongst samples were displayed based on the Bray-Curtis similarity between fungal communities. The values for all samples were square root transformed. (TIF) [file pone.0179855.s010.tif]

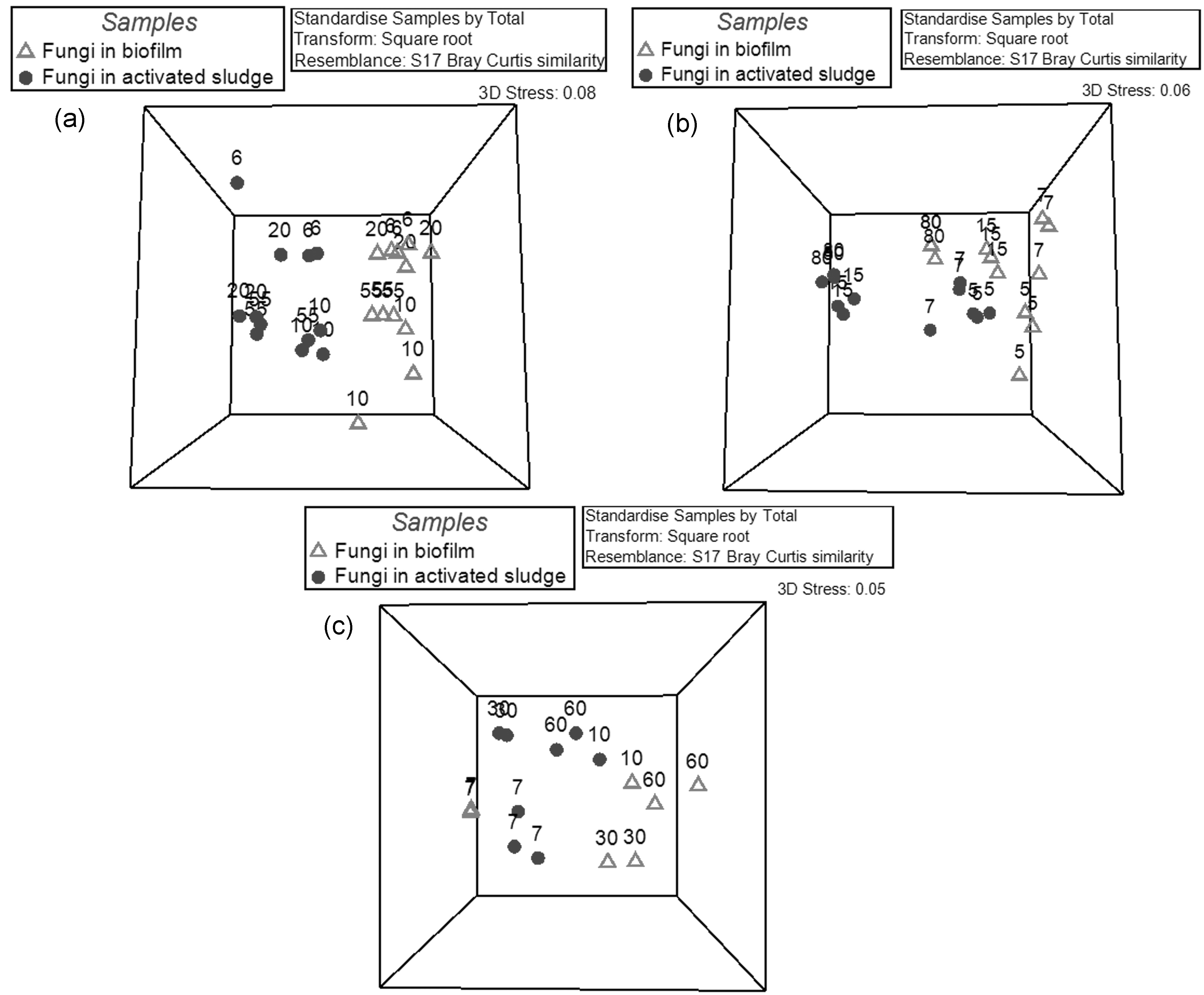

Supplement: S11 Fig — The blue circles represent the fungal communities in the sludge, and the empty triangles represent the fungal communities in the biofilms. The data for all the samples was the OTUs of 18S rDNA at cutoff of 0.03. The numbers in the plots represent the TMP values (kPa) when the samples were collected. The relationships amongst samples were displayed based on the Bray-Curtis similarity between fungal communities. The values for all samples were square root transformed. (TIF) [file pone.0179855.s011.tif]

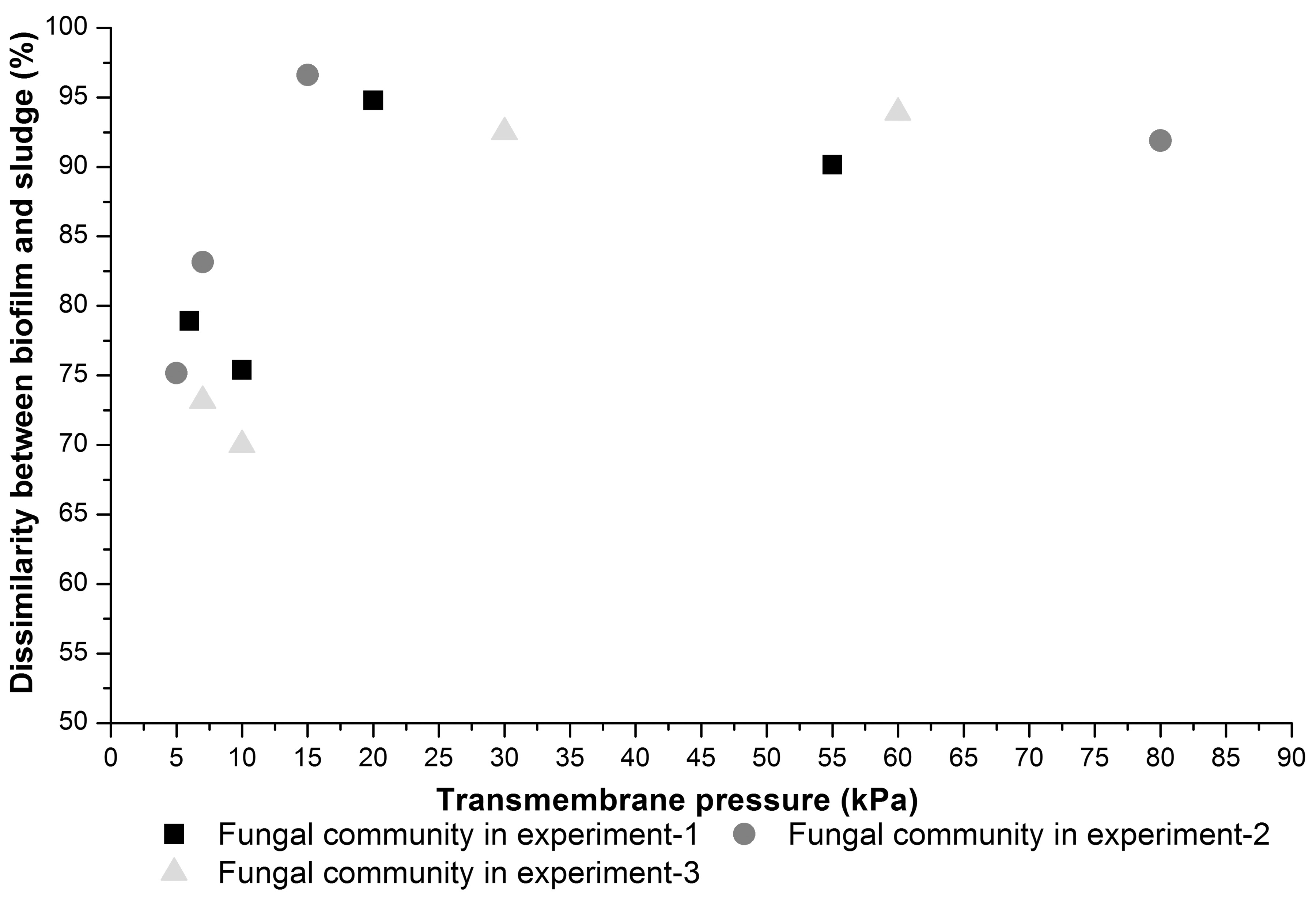

Supplement: S12 Fig — The dissimilarity values were calculated from the biofilm and sludge samples at the same TMP through the “SIMPER” process in PRIMER v6. (TIF) [file pone.0179855.s012.tif]
