# Supplementary material for: Succession of biofilm communities responsible for biofouling of membrane bio-reactors (MBRs)
Source: PLoS One. 2017 Jul 7;12(7):e0179855. doi: 10.1371/journal.pone.0179855 (PMC5501448; doi:10.1371/journal.pone.0179855)
Supplement: S1 Table — (DOCX) [file pone.0179855.s013.docx]

**S1 Table** The primers and probes used in this study.

| Name | Sequence (5’ – 3’) and modifications | Function | Target | Reference |
| --- | --- | --- | --- | --- |
| ARC787F | ATTAGATACCCSBGTAGTCC | qPCR | Archaea | (Lee et al. 2008) |
| ARC1059R | GCCAT GCACC WCCTC T | qPCR | Archaea |  |
| ARC915F | (6FAM) – AGGAATTGGCGGGGGAGCAC–(BBQ) | qPCR | Archaea |  |
| BAC338F | ACTCCTACGGGAGGCAG | qPCR | Bacteria | (Shin et al. 2010) |
| BAC805R | GACTACCAGGGTATCTAATCC | qPCR | Bacteria |  |
| BAC516F | (6FAM) –TGCCAGCAGCCGCGGTAATAC–(BBQ) | qPCR | Bacteria |  |
| FungiQuant-F | GGRAAACTCACCAGGTCCAG | qPCR | Fungi | (Liu et al. 2012) |
| FungiQuant-R | GSWCTATCCCCAKCACGA | qPCR | Fungi |  |
| FungiQuant-Prb^LNA^ | (6FAM) –TGGTGCATGG+C+CGTT–(BBQ) | qPCR | Fungi |  |
| Gray28F | GAGTTTGATCNTGGCTCAG | Sequencing | Bacteria | (Cytryn et al. 2012) |
| Gray519R | GTNTTACNGCGGCKGCTG | Sequencing | Bacteria |  |
| funSSUF | TGGAGGGCAAGTCTGGTG | Sequencing | Fungi | (Foster et al. 2013) |
| funSSUR | TCGGCATAGTTTATGGTTAAG | Sequencing | Fungi |  |
