# Supplementary material for: Succession of biofilm communities responsible for biofouling of membrane bio-reactors (MBRs)
Source: PLoS One. 2017 Jul 7;12(7):e0179855. doi: 10.1371/journal.pone.0179855 (PMC5501448; doi:10.1371/journal.pone.0179855)
Supplement: S2 Table — (DOCX) [file pone.0179855.s014.docx]

**S2 Table** The accession numbers of the sequence packets in Genbank in the 3 experiments.

| **Sequence packets** | **Accession No. in the Genbank** |
| --- | --- |
| Bacterial sequence packets in experiment-1 | SRR2177521, SRR2177523, SRR3199053, SRR3199112,  SRR3199113, SRR3199235, SRR3199236, SRR3199237,  SRR3199238, SRR3199239, SRR3199240, SRR3199241  SRR3199242, SRR3199244, SRR3199245, SRR3199246,  SRR3199247, SRR3199248, SRR3199250, SRR3199251,  SRR3199252, SRR3199253, SRR3201596, SRR3201599 |
| Bacterial sequence packets in experiment-2 | SRR3201601, SRR3201615, SRR3201618, SRR3201620,  SRR3201622, RR3201626, SRR3201627, SRR3201628,  SRR3201637, SRR3201638, SRR3201643, SRR3201645,  SRR3201647, SRR3201648, SRR3201649, SRR3201662,  SRR3201663, SRR3201664, SRR3201666, SRR3201689,  SRR3201705, SRR3201711, SRR3201728, SRR3201730 |
| Bacterial sequence packets in experiment-3 | SRR3201732, SRR3201733, SRR3201734, SRR3201735,  SRR3201736, SRR3201737, SRR3201738, SRR3201739,  SRR3201740, SRR3201741, SRR3201742, SRR3201743,  SRR3201744, SRR3201746, SRR3201747, SRR3201748,  SRR3201749 |
| Fungal sequence packets in experiment-1 | SRR3203090, SRR3203091, SRR3203092, SRR3203093,  SRR3203094, SRR3203095, SRR3203097, SRR3203098,  SRR3203099, SRR3203101, SRR3203102, SRR3203103,  SRR3203128, SRR3203129, SRR3203130, SRR3203133,  SRR3203134, SRR3203135, SRR3203137, SRR3203138,  SRR3203141, SRR3203142, SRR3203143, SRR3203145 |
| Fungal sequence packets in experiment-2 | SRR3203146, SRR3203149, SRR3203156, SRR3203159,  SRR3203161, SRR3203162, SRR3203170, SRR3203171,  SRR3203173, SRR3203177, SRR3203180, SRR3203184,  SRR3203207, SRR3203208, SRR3203209, SRR3203210,  SRR3203211, SRR3203213, SRR3203215, SRR3203216,  SRR3203274, SRR3203275, SRR3203276, SRR3203277 |
| Fungal sequence packets in experiment-3 | SRR3203279, SRR3203280, SRR3203282, SRR3203283,  SRR3203284, SRR3203285, SRR3203287, SRR3203288,  SRR3203289, SRR3203290, SRR3203291, SRR3203292,  SRR3203294, SRR3203295, SRR3203296, SRR3203297 |
