# Supplementary material for: Succession of biofilm communities responsible for biofouling of membrane bio-reactors (MBRs)
Source: PLoS One. 2017 Jul 7;12(7):e0179855. doi: 10.1371/journal.pone.0179855 (PMC5501448; doi:10.1371/journal.pone.0179855)
Supplement: S3 Table — (DOCX) [file pone.0179855.s015.docx]

**S3 Table The dominant bacterial OTU s in biofilms at the low TMP in the 3 replicate experiments** (a)

| Dominant OTUs in experiment-1 | | Taxonomic classification | | |
| --- | --- | --- | --- | --- |
| OTUs at 6kPa | Abundance (%) | Order | Family | Genus |
| OTU 4 | 0.52 | Actinomycetales | Kineosporiaceae | unclassified |
| OTU 84 | 0.88 | Burkholderiales | Burkholderiaceae | *Ralstonia* |
| OTU 36 | 0.8 |  |  | *Cupriavidus* |
| OTU 37 | 1.6 |  | Comamonadaceae | *Pelomonas* |
| OTU 12 | 1.47 |  |  | *Ideonella* |
| OTU 27 | 0.88 |  |  |  |
| OTU 88 | 0.85 |  |  |  |
| OTU 145 | 0.9 |  |  | unclassified |
| OTU 16 | 0.71 |  |  | unclassified |
| OTU 19 | 1.42 | Pseudomonadales | Moraxellaceae | *Perlucidibaca* |
| OTU 38 | 1.08 |  |  |  |
| OTU 41 | 1.01 |  |  |  |
| OTU 118 | 0.57 |  |  |  |
| OTU 123 | 0.52 |  |  |  |
| OTU 116 | 0.43 |  |  |  |
| OTU 2 | 1.55 | Rhodocyclales | Rhodocyclaceae | *Zoogloea* |
| OTU 5 | 0.97 |  |  |  |
| OTU 22 | 0.52 |  |  |  |
| OTU 73 | 1.08 | Sphingobacteriales | Cytophagaceae | *Cytophaga* |
| OTU 7 | 4.09 |  |  | *Flexibacter* |
| OTU 15 | 0.82 |  |  |  |
| OTU 10 | 0.72 |  |  |  |
| OTU 17 | 0.6 |  |  |  |
| OTU 44 | 1.19 |  |  | unclassified |
| OTU 30 | 0.87 |  | env.OPS_17 | unclassified |
| OTU 1 | 1.94 |  | unclassified | unclassified |
| OTU 32 | 1.55 |  | unclassified | unclassified |
| OTU 112 | 0.59 |  | unclassified | unclassified |
| OTU 164 | 0.47 |  | unclassified | unclassified |
| OTU 175 | 0.47 |  | unclassified | unclassified |
| OTU 111 | 0.58 | Xanthomonadales | Xanthomonadaceae | *Stenotrophomonas* |
| OTU 63 | 0.6 | 12up | unclassified | unclassified |

(b)

| Dominant OTUs in experiment-2 | | Taxonomic classification | | |
| --- | --- | --- | --- | --- |
| OTUs at 5kPa | Abundance (%) | Order | Family | Genus |
| OTU 57 | 0.76 | Burkholderiales | Burkholderiaceae | *Cupriavidus* |
| OTU 120 | 0.9 |  | Comamonadaceae | *Pelomonas* |
| OTU 187 | 0.54 |  |  |  |
| OTU 191 | 0.64 |  |  |  |
| OTU 230 | 0.49 |  |  |  |
| OTU 7 | 0.66 | Opitutales | Opitutaceae | *Opitutus* |
| OTU 17 | 4.13 |  |  |  |
| OTU 63 | 1.05 |  |  |  |
| OTU 72 | 1.65 |  |  |  |
| OTU 123 | 1.1 |  |  |  |
| OTU 243 | 0.82 |  |  |  |
| OTU 74 | 1.01 | Planctomycetales | Planctomycetaceae | *Planctomyces* |
| OTU 6 | 0.69 | Rhodocyclales | Rhodocyclaceae | *Zoogloea* |
| OTU 24 | 3.28 |  |  |  |
| OTU 29 | 2.6 |  |  |  |
| OTU 73 | 1.47 |  |  |  |
| OTU 76 | 0.54 |  |  |  |
| OTU 79 | 1.66 |  |  |  |
| OTU 82 | 1.44 |  |  |  |
| OTU 185 | 0.6 |  |  |  |
| OTU 200 | 0.51 |  |  |  |
| OTU 289 | 0.5 |  |  |  |
| OTU 311 | 0.55 |  |  |  |
| OTU 353 | 0.53 |  |  |  |
| OTU 397 | 0.65 |  |  |  |
| OTU 124 | 0.96 | Sphingobacteriales | Saprospiraceae | unclassified |
| OTU 202 | 0.79 |  | unclassified | unclassified |
| OTU 3 | 0.68 |  | unclassified | unclassified |
| OTU 9 | 0.57 |  | unclassified | unclassified |
| OTU 18 | 0.93 |  | unclassified | unclassified |
| OTU 68 | 0.6 |  | unclassified | unclassified |
| OTU 212 | 0.72 |  | unclassified | unclassified |

(c)

| Dominant OTUs in experiment-3 | | Taxonomic classification | | |
| --- | --- | --- | --- | --- |
| OTUs at 7kPa | Abundance (%) | Order | Family | Genus |
| OTU 116 | 1.52 | Acidobacteriales | Acidobacteriaceae | Candidatus*_*  *Chloroacidobacterium* |
| OTU 36 | 0.86 | Actinomycetales | Propionibacteriaceae | *Micropruina* |
| OTU 56 | 0.69 |  | Microbacteriaceae | *Leucobacter* |
| OTU 114 | 0.54 | Burkholderiales | Comamonadaceae | *Rubrivivax* |
| OTU 214 | 0.51 |  |  | unclassified |
| OTU 89 | 0.56 | Rhizobiales | Methylobacteriaceae | *Meganema* |
| OTU 1 | 1.81 | Rhodocyclales | Rhodocyclaceae | *Zoogloea* |
| OTU 4 | 1.33 |  |  |  |
| OTU 19 | 0.67 |  |  |  |
| OTU 13 | 0.65 |  |  |  |
| OTU 3 | 1.98 | Sphingobacteriales | Chitinophagaceae | uncultured |
| OTU 165 | 0.69 |  |  | unclassified |
| OTU 359 | 0.51 |  |  | unclassified |
| OTU 61 | 0.79 |  | Cytophagaceae | *Runella* |
| OTU 126 | 0.51 |  | Saprospiraceae | uncultured |
| OTU 104 | 0.95 |  |  |  |
| OTU 34 | 0.65 |  | Sphingomonadaceae | *Sphingopyxis* |
| OTU 173 | 1.07 |  | unclassified | unclassified |
| OTU 78 | 1.01 |  | unclassified | unclassified |
| OTU 132 | 0.66 |  | unclassified | unclassified |
| OTU 381 | 0.57 |  | unclassified | unclassified |
| OTU 2 | 0.89 | Thiotrichales | Thiotrichaceae | *Thiothrix* |
