# Supplementary material for: Succession of biofilm communities responsible for biofouling of membrane bio-reactors (MBRs)
Source: PLoS One. 2017 Jul 7;12(7):e0179855. doi: 10.1371/journal.pone.0179855 (PMC5501448; doi:10.1371/journal.pone.0179855)
Supplement: S4 Table — (DOCX) [file pone.0179855.s016.docx]

**S4 Table** The dominant bacterial OTUs in biofilms at the high TMP in the 3 replicate experiments

(a)

| Dominant OTUs in experiment-1 | | Taxonomic classification | | |
| --- | --- | --- | --- | --- |
| OTUs at 55 kPa | Abundance (%) | Order | Family | Genus |
| OTU 4 | 1.57 | Actinomycetales | Kineosporiaceae | unclassified |
| OTU 6 | 1.78 | Burkholderiales | Comamonadaceae | unclassified |
| OTU 3 | 1.6 |  |  | unclassified |
| OTU 16 | 0.89 |  |  | unclassified |
| OTU 203 | 0.69 |  |  | unclassified |
| OTU 68 | 0.49 | Lactobacillales | Streptococcaceae | *Lactococcus* |
| OTU 135 | 0.6 | Myxococcales | Haliangiaceae | *Haliangium* |
| OTU 92 | 0.59 |  | Polyangiaceae | *Sorangium* |
| OTU 8 | 1.12 | Nitrospirales | Nitrospiraceae | *Nitrospira* |
| OTU 57 | 0.64 | Planctomycetales | Planctomycetaceae | *Planctomyces* |
| OTU 2 | 0.86 | Rhodocyclales | Rhodocyclaceae | *Zoogloea* |
| OTU 5 | 0.52 |  |  |  |
| OTU 1 | 3.12 | Sphingobacteriales | unclassified | unclassified |
| OTU 15 | 1.37 |  | Cytophagaceae | *Flexibacter* |
| OTU 10 | 1.01 |  |  |  |
| OTU 17 | 0.89 |  |  |  |
| OTU 48 | 0.57 |  |  |  |
| OTU 18 | 1.35 |  | Sphingomonadaceae | *Sphingopyxis* |
| OTU 26 | 0.92 |  |  |  |
| OTU 29 | 0.82 |  |  |  |
| OTU 80 | 0.75 |  |  | *Sphingomonas* |
| OTU 64 | 1.03 | Xanthomonadales | Xanthomonadaceae | unclassified |

(b)

| Dominant OTUs in experiment-2 | | Taxonomic classification | | |
| --- | --- | --- | --- | --- |
| OTUs at 80 kPa | Abundance (%) | Order | Family | Genus |
| OTU 4 | 1.8 | Burkholderiales | Comamonadaceae | *Ideonella* |
| OTU 5 | 1.61 |  |  |  |
| OTU 10 | 1.15 |  |  |  |
| OTU 12 | 1.03 |  |  |  |
| OTU 21 | 0.83 |  |  |  |
| OTU 14 | 0.79 |  |  |  |
| OTU 32 | 0.66 |  |  |  |
| OTU 37 | 0.59 |  |  |  |
| OTU 35 | 0.55 |  |  |  |
| OTU 39 | 0.48 | Flavobacteriales | Flavobacteriaceae | unclassified |
| OTU 7 | 0.48 | Opitutales | Opitutaceae | *Opitutus* |
| OTU 41 | 0.48 |  |  |  |
| OTU 2 | 2.86 | Sphingobacteriales | unclassified | unclassified |
| OTU 1 | 2.57 |  | unclassified | unclassified |
| OTU 28 | 0.9 |  | unclassified | unclassified |
| OTU 26 | 0.61 |  | unclassified | unclassified |
| OTU 9 | 0.58 |  | unclassified | unclassified |
| OTU 45 | 0.53 |  | unclassified | unclassified |
| OTU 27 | 0.49 |  | env.OPS_17 | unclassified |
| OTU 16 | 0.65 |  | env.OPS_17 | unclassified |

(c)

| Dominant OTUs in experiment-3 | | Taxonomic classification | | |
| --- | --- | --- | --- | --- |
| OTUs at 60 kPa | Abundance (%) | Order | Family | Genus |
| OTU 40 | 2.55 | Burkholderiales | Comamonadaceae | *Pelomonas* |
| OTU 16 | 1.45 |  |  |  |
| OTU 121 | 1.15 |  |  | unclassified |
| OTU 176 | 0.71 |  |  | *Ideonella* |
| OTU 32 | 0.45 |  |  | unclassified |
| OTU 87 | 0.5 | Lactobacillales | Streptococcaceae | *Lactococcus* |
| OTU 62 | 0.72 | Pseudomonadales | Pseudomonadaceae | *Cellvibrio* |
| OTU 91 | 0.69 |  |  |  |
| OTU 1 | 0.83 | Rhodocyclales | Rhodocyclaceae | *Zoogloea* |
| OTU 4 | 0.42 |  |  |  |
| OTU 6 | 15.01 | Sphingobacteriales | Cytophagaceae | *Cytophaga* |
| OTU 17 | 4.51 |  |  |  |
| OTU 191 | 0.64 |  |  |  |
| OTU 3 | 3.68 |  | Chitinophagaceae | uncultured |
| OTU 34 | 1.82 |  | Sphingomonadaceae | *Sphingopyxis* |
| OTU 72 | 0.84 |  |  |  |
| OTU 2 | 0.56 | Thiotrichales | Thiotrichaceae | *Thiothrix* |
