# Supplementary material for: Succession of biofilm communities responsible for biofouling of membrane bio-reactors (MBRs)
Source: PLoS One. 2017 Jul 7;12(7):e0179855. doi: 10.1371/journal.pone.0179855 (PMC5501448; doi:10.1371/journal.pone.0179855)
Supplement: S5 Table — (DOCX) [file pone.0179855.s017.docx]

**S5 Table** the dominant fungal OTUs in the early biofilms at low TMP in experiment-1, 2 and 3.

(a)

| OTUs at  6 kPa | Average abundance (%) | Contribution to the  group similarity (%) | Taxonomic classification | | | |
| --- | --- | --- | --- | --- | --- | --- |
|  |  |  | Kingdom | Subkingdom | Phylum | Genus |
| OTU 10 | 7.3 | 11.48 | Fungi | Dikarya | unclassified | unclassified |
| OTU 11 | 5.93 | 8.31 | Fungi | unclassified | unclassified | unclassified |
| OTU 27 | 3.28 | 4.88 | Fungi | Dikarya | unclassified | unclassified |
| OTU 24 | 3.41 | 4.48 | Fungi | Dikarya | unclassified | unclassified |
| OTU 3 | 2.36 | 3.39 | Fungi | Dikarya | Ascomycota | *Candida* |
| OTU 2 | 3.4 | 3.13 | Fungi | unclassified | unclassified | unclassified |
| OTU 14 | 2.09 | 2.46 | Fungi | unclassified | unclassified | unclassified |
| OTU 67 | 1.47 | 2.08 | Fungi | Dikarya | unclassified | unclassified |
| OTU 37 | 2.07 | 2.01 | Fungi | unclassified | unclassified | unclassified |
| OTU 89 | 1.1 | 1.8 | Fungi | Dikarya | unclassified | unclassified |
| OTU 94 | 1.26 | 1.73 | Fungi | Dikarya | Ascomycota | unclassified |
| OTU 63 | 1.39 | 1.69 | Fungi | Dikarya | unclassified | unclassified |
| OTU 12 | 1.44 | 1.55 | Fungi | unclassified | unclassified | unclassified |
| OTU 15 | 1.24 | 1.55 | Fungi | Dikarya | Ascomycota | unclassified |
| OTU 92 | 1.02 | 1.5 | Fungi | Dikarya | unclassified | unclassified |
| OTU 86 | 1.36 | 1.45 | Fungi | Dikarya | unclassified | unclassified |
| OTU 4 | 2.6 | 1.36 | Fungi | Dikarya | Basidiomycota | unclassified |
| OTU 5 | 0.8 | 1.27 | Fungi | unclassified | unclassified | unclassified |
| OTU 79 | 1.03 | 1.26 | Fungi | unclassified | unclassified | unclassified |
| OTU 1 | 0.89 | 1.25 | Fungi | Dikarya | unclassified | unclassified |
| OTU 9 | 1.46 | 1.15 | Fungi | Dikarya | unclassified | unclassified |
| OTU 97 | 0.9 | 1.15 | Fungi | Dikarya | unclassified | unclassified |
| OTU 105 | 0.98 | 1.09 | Fungi | Dikarya | unclassified | unclassified |
| OTU 132 | 0.72 | 1.05 | Fungi | Dikarya | unclassified | unclassified |
| OTU 17 | 0.93 | 1 | Fungi | unclassified | unclassified | unclassified |
| OTU 19 | 0.79 | 0.98 | Fungi | Dikarya | Ascomycota | *Candida* |
| OTU 69 | 1.24 | 0.92 | Fungi | unclassified | unclassified | unclassified |
| OTU 144 | 0.71 | 0.89 | Fungi | Dikarya | unclassified | unclassified |
| OTU 164 | 0.63 | 0.89 | Fungi | Dikarya | unclassified | unclassified |
| OTU 109 | 0.76 | 0.8 | Fungi | Dikarya | unclassified | unclassified |
| OTU 49 | 0.6 | 0.79 | Fungi | Dikarya | unclassified | unclassified |
| OTU 150 | 0.62 | 0.69 | Fungi | unclassified | unclassified | unclassified |
| OTU 85 | 0.58 | 0.65 | Fungi | Dikarya | unclassified | unclassified |
| OTU 38 | 0.48 | 0.64 | Fungi | Dikarya | unclassified | unclassified |
| OTU 6 | 1 | 0.62 | Fungi | Dikarya | unclassified | unclassified |
| OTU 40 | 0.57 | 0.58 | Fungi | unclassified | unclassified | unclassified |

**(b)**

| OTUs at  5 kPa | Average abundance (%) | Contribution to the  group similarity (%) | Taxonomic classification | | | |
| --- | --- | --- | --- | --- | --- | --- |
|  |  |  | Kingdom | Subkingdom | Phylum | Genus |
| OTU 1 | 9.31 | 14.35 | Fungi | Dikarya | unclassified | unclassified |
| OTU 4 | 4.66 | 10.78 | Fungi | unclassified | unclassified | unclassified |
| OTU 10 | 3.49 | 7.82 | Fungi | Dikarya | unclassified | unclassified |
| OTU 5 | 4.3 | 5.98 | Fungi | Dikarya | Ascomycota | *Candida* |
| OTU 27 | 1.59 | 2.94 | Fungi | unclassified | unclassified | unclassified |
| OTU 9 | 2.92 | 2.51 | Fungi | unclassified | unclassified | unclassified |
| OTU 42 | 4.17 | 2.39 | Fungi | Dikarya | Basidiomycota | unclassified |
| OTU 6 | 2.18 | 2.19 | Fungi | Dikarya | unclassified | unclassified |
| OTU 19 | 1.84 | 2.05 | Fungi | Dikarya | Ascomycota | *Candida* |
| OTU 13 | 1.22 | 1.87 | Fungi | unclassified | unclassified | unclassified |
| OTU 20 | 0.96 | 1.58 | Fungi | Dikarya | unclassified | unclassified |
| OTU 34 | 1.58 | 1.57 | Fungi | Dikarya | unclassified | unclassified |
| OTU 16 | 0.75 | 1.35 | Fungi | Dikarya | unclassified | unclassified |
| OTU 53 | 0.73 | 1.32 | Fungi | unclassified | unclassified | unclassified |
| OTU 14 | 0.8 | 1.24 | Fungi | Dikarya | unclassified | unclassified |
| OTU 74 | 0.53 | 1.21 | Fungi | Dikarya | unclassified | unclassified |
| OTU 49 | 0.54 | 1.17 | Fungi | Dikarya | Ascomycota | *Candida* |
| OTU 18 | 0.57 | 0.94 | Fungi | Dikarya | unclassified | unclassified |
| OTU 12 | 1.54 | 0.91 | Fungi | Dikarya | unclassified | unclassified |

**(c)**

| OTUs at  7 kPa | Average abundance (%) | Contribution to the  group similarity (%) | Kingdom | Subkingdom | Phylum | Genus |
| --- | --- | --- | --- | --- | --- | --- |
| OTU 1 | 7.77 | 10.46 | Fungi | Dikarya | unclassified | unclassified |
| OTU 2 | 6.35 | 8.28 | Fungi | unclassified | unclassified | unclassified |
| OTU 5 | 3.85 | 4.4 | Fungi | Dikarya | unclassified | unclassified |
| OTU 4 | 4.67 | 4 | Fungi | Dikarya | unclassified | unclassified |
| OTU 8 | 2.87 | 3.86 | Fungi | Dikarya | unclassified | unclassified |
| OTU 12 | 2.35 | 3.5 | Fungi | Dikarya | unclassified | unclassified |
| OTU 10 | 2.24 | 3.14 | Fungi | Dikarya | unclassified | unclassified |
| OTU 6 | 2.6 | 2.98 | Fungi | Dikarya | unclassified | unclassified |
| OTU 11 | 2.29 | 2.77 | Fungi | Dikarya | unclassified | unclassified |
| OTU 3 | 3.74 | 2.67 | Fungi | unclassified | unclassified | unclassified |
| OTU 7 | 2.54 | 2.66 | Fungi | Dikarya | unclassified | unclassified |
| OTU 18 | 1.66 | 2.25 | Fungi | Dikarya | unclassified | unclassified |
| OTU 13 | 1.11 | 1.31 | Fungi | unclassified | unclassified | unclassified |
| OTU 19 | 1.55 | 1.27 | Fungi | Dikarya | unclassified | unclassified |
| OTU 16 | 1.61 | 1.22 | Fungi | unclassified | unclassified | unclassified |
| OTU 24 | 1.02 | 1.2 | Fungi | Dikarya | unclassified | unclassified |
| OTU 20 | 1.21 | 1.17 | Fungi | Dikarya | unclassified | unclassified |
| OTU 26 | 0.81 | 0.98 | Fungi | Dikarya | unclassified | unclassified |
| OTU 29 | 0.79 | 0.87 | Fungi | Dikarya | unclassified | unclassified |
| OTU 14 | 0.52 | 0.82 | Fungi | Glomeromycota | Glomeromycetes | unclassified |
| OTU 39 | 0.56 | 0.54 | Fungi | Dikarya | unclassified | unclassified |
| OTU 25 | 0.66 | 0.54 | Fungi | Dikarya | unclassified | unclassified |
| OTU 28 | 0.69 | 0.53 | Fungi | Dikarya | unclassified | unclassified |
