# Supplementary material for: Succession of biofilm communities responsible for biofouling of membrane bio-reactors (MBRs)
Source: PLoS One. 2017 Jul 7;12(7):e0179855. doi: 10.1371/journal.pone.0179855 (PMC5501448; doi:10.1371/journal.pone.0179855)
Supplement: S6 Table — (DOCX) [file pone.0179855.s018.docx]

**S6 Table** The dominant fungal OTUs in the late biofilms at high TMP in experiment-1, 2 and 3.

(a)

| OTUs at  55 kPa | Average abundance (%) | Contribution to the  group similarity (%) | Kingdom | Subkingdom | Phylum | Genus |
| --- | --- | --- | --- | --- | --- | --- |
| OTU 1 | 15.75 | 20.66 | Fungi | Dikarya | unclassified | unclassified |
| OTU 2 | 6.77 | 8.55 | Fungi | unclassified | unclassified | unclassified |
| OTU 3 | 4.53 | 5.52 | Fungi | Dikarya | Ascomycota | *Candida* |
| OTU 12 | 3.21 | 4.18 | Fungi | unclassified | unclassified | unclassified |
| OTU 13 | 2.73 | 3.68 | Fungi | Dikarya | unclassified | unclassified |
| OTU 17 | 2.07 | 2.73 | Fungi | unclassified | unclassified | unclassified |
| OTU 16 | 2.08 | 2.61 | Fungi | unclassified | unclassified | unclassified |
| OTU 21 | 1.79 | 2.19 | Fungi | Dikarya | unclassified | unclassified |
| OTU 19 | 1.35 | 1.8 | Fungi | Dikarya | Ascomycota | *Candida* |
| OTU 29 | 1.32 | 1.72 | Fungi | Dikarya | unclassified | unclassified |
| OTU 28 | 1.16 | 1.69 | Fungi | Dikarya | Ascomycota | *Metschnikowia* |
| OTU 22 | 1.34 | 1.63 | Fungi | unclassified | unclassified | unclassified |
| OTU 35 | 1.31 | 1.62 | Fungi | Dikarya | unclassified | unclassified |
| OTU 25 | 1.22 | 1.59 | Fungi | Dikarya | Ascomycota | *Candida* |
| OTU 40 | 0.89 | 1.21 | Fungi | unclassified | unclassified | unclassified |
| OTU 18 | 1.22 | 1.19 | Fungi | Dikarya | Ascomycota | *Candida* |
| OTU 45 | 0.75 | 1.01 | Fungi | unclassified | unclassified | unclassified |
| OTU 32 | 0.82 | 0.99 | Fungi | Dikarya | Ascomycota | *Candida* |
| OTU 54 | 0.75 | 0.95 | Fungi | unclassified | unclassified | unclassified |
| OTU 47 | 0.77 | 0.86 | Fungi | Glomeromycota | Glomeromycetes | unclassified |
| OTU 59 | 0.72 | 0.84 | Fungi | unclassified | unclassified | unclassified |
| OTU 42 | 0.7 | 0.77 | Fungi | Dikarya | Ascomycota | *Candida* |
| OTU 62 | 0.51 | 0.72 | Fungi | unclassified | unclassified | unclassified |

(b)

| OTUs at 80 kPa | Average abundance (%) | Contribution to the  group similarity (%) | Kingdom | Subkingdom | Phylum | Genus |
| --- | --- | --- | --- | --- | --- | --- |
| OTU 8 | 10.63 | 14.76 | Fungi | Dikarya | Ascomycota | unclassified |
| OTU 3 | 6.72 | 9.49 | Fungi | unclassified | unclassified | unclassified |
| OTU 15 | 6.29 | 8.42 | Fungi | Dikarya | Ascomycota | unclassified |
| OTU 22 | 4.1 | 4.49 | Fungi | Dikarya | Ascomycota | unclassified |
| OTU 11 | 2.82 | 3.81 | Fungi | unclassified | unclassified | unclassified |
| OTU 17 | 2.28 | 2.92 | Fungi | unclassified | unclassified | unclassified |
| OTU 29 | 1.79 | 2.33 | Fungi | unclassified | unclassified | unclassified |
| OTU 63 | 1.78 | 1.42 | Fungi | Dikarya | Ascomycota | unclassified |
| OTU 1 | 1.46 | 1.39 | Fungi | Dikarya | unclassified | unclassified |
| OTU 66 | 1.11 | 1.26 | Fungi | unclassified | unclassified | unclassified |
| OTU 122 | 0.79 | 1.15 | Fungi | Dikarya | Ascomycota | unclassified |
| OTU 37 | 0.91 | 1.07 | Fungi | unclassified | unclassified | unclassified |
| OTU 4 | 0.82 | 1.07 | Fungi | unclassified | unclassified | unclassified |
| OTU 59 | 0.66 | 0.91 | Fungi | unclassified | unclassified | unclassified |
| OTU 50 | 0.69 | 0.91 | Fungi | unclassified | unclassified | unclassified |
| OTU 55 | 0.68 | 0.87 | Fungi | unclassified | unclassified | unclassified |
| OTU 156 | 0.62 | 0.86 | Fungi | Dikarya | Ascomycota | unclassified |
| OTU 39 | 0.83 | 0.82 | Fungi | Dikarya | Ascomycota | *Candida* |
| OTU 69 | 0.53 | 0.81 | Fungi | unclassified | unclassified | unclassified |
| OTU 161 | 0.58 | 0.79 | Fungi | Dikarya | unclassified | unclassified |
| OTU 62 | 0.54 | 0.77 | Fungi | unclassified | unclassified | unclassified |
| OTU 118 | 0.72 | 0.75 | Fungi | Dikarya | Ascomycota | *Metschnikowia* |
| OTU 107 | 0.56 | 0.75 | Fungi | Dikarya | Ascomycota | unclassified |

(c)

| OTUs at  60 kPa | Average abundance (%) | Contribution to the  group similarity (%) | Kingdom | Subkingdom | Phylum | Genus |
| --- | --- | --- | --- | --- | --- | --- |
| OTU 35 | 1.5 | 6.92 | Fungi | unclassified | unclassified | unclassified |
| OTU 31 | 4.47 | 4.15 | Fungi | unclassified | unclassified | unclassified |
| OTU 131 | 1.09 | 4.15 | Fungi | unclassified | unclassified | unclassified |
| OTU 176 | 0.94 | 4.15 | Fungi | unclassified | unclassified | unclassified |
| OTU 325 | 0.39 | 4.15 | Fungi | unclassified | unclassified | unclassified |
| OTU 50 | 2.9 | 2.77 | Fungi | unclassified | unclassified | unclassified |
| OTU 66 | 2.24 | 2.77 | Fungi | unclassified | unclassified | unclassified |
| OTU 78 | 1.07 | 2.77 | Fungi | unclassified | unclassified | unclassified |
| OTU 89 | 1.44 | 2.77 | Fungi | unclassified | unclassified | unclassified |
| OTU 92 | 0.45 | 2.66 | Fungi | Dikarya | unclassified | unclassified |
| OTU 81 | 1.51 | 1.77 | Fungi | Dikarya | Ascomycota | *Candida* |
| OTU 100 | 0.7 | 1.77 | Fungi | Dikarya | Ascomycota | *Candida* |
| OTU 49 | 0.9 | 1.38 | Fungi | Glomeromycota | Glomeromycetes | unclassified |
| OTU 73 | 1.67 | 1.38 | Fungi | unclassified | unclassified | unclassified |
| OTU 135 | 0.75 | 1.38 | Fungi | unclassified | unclassified | unclassified |
| OTU 200 | 0.61 | 1.38 | Fungi | unclassified | unclassified | unclassified |
| OTU 201 | 0.57 | 1.38 | Fungi | unclassified | unclassified | unclassified |
| OTU 203 | 0.61 | 1.38 | Fungi | unclassified | unclassified | unclassified |
